# Multidimensional population activity in an electrically coupled inhibitory circuit in the cerebellar cortex

**DOI:** 10.1016/j.neuron.2021.03.027

**Published:** 2021-05-19

**Authors:** Harsha Gurnani, R. Angus Silver

**Affiliations:** 1 Department of Neuroscience, Physiology, and Pharmacology, University College London, London WC1E 6BT, UK

**Keywords:** inhibitory interneurons, population codes, gap junctions, electrical coupling, Golgi cells, cerebellar cortex, dimensionality, inhibition, gain control

## Abstract

Inhibitory neurons orchestrate the activity of excitatory neurons and play key roles in circuit function. Although individual interneurons have been studied extensively, little is known about their properties at the population level. Using random-access 3D two-photon microscopy, we imaged local populations of cerebellar Golgi cells (GoCs), which deliver inhibition to granule cells. We show that population activity is organized into multiple modes during spontaneous behaviors. A slow, network-wide common modulation of GoC activity correlates with the level of whisking and locomotion, while faster (<1 s) differential population activity, arising from spatially mixed heterogeneous GoC responses, encodes more precise information. A biologically detailed GoC circuit model reproduced the common population mode and the dimensionality observed experimentally, but these properties disappeared when electrical coupling was removed. Our results establish that local GoC circuits exhibit multidimensional activity patterns that could be used for inhibition-mediated adaptive gain control and spatiotemporal patterning of downstream granule cells.

## Highlights

• Inhibitory circuit population activity is organized on multiple spatiotemporal scales
• Slow circuit-wide Golgi cell activation reflects general level of behavioral activity
• Multidimensional differential population activity encodes behavioral information
• Electrically coupled Golgi cell circuit model reproduces population-level properties

## Graphical abstract

**Figure:**
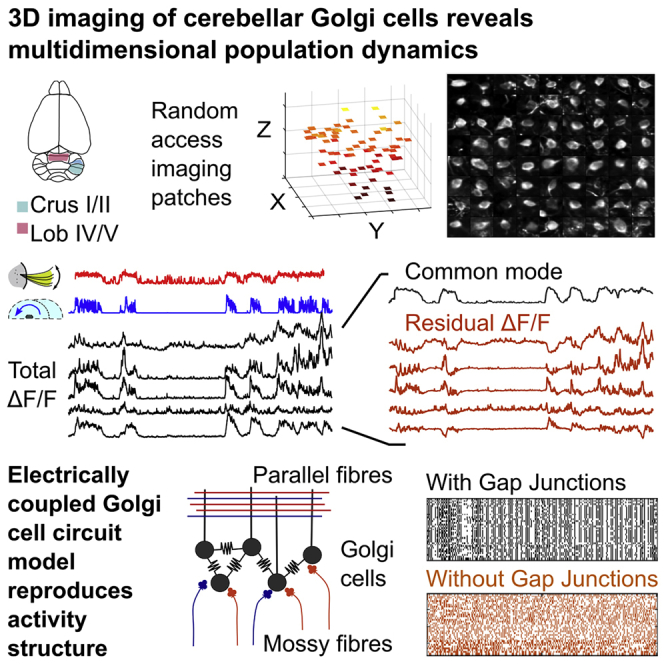

## Teaser

Inhibitory interneurons orchestrate the activity of neural circuits, but little is known about their population dynamics. By using 3D random-access calcium imaging, Gurnani and Silver show that cerebellar Golgi cell circuits exhibit multidimensional activity with common and distributed modes. A biologically detailed circuit model implicates electrical coupling in shaping the population dynamics.

## Introduction

Inhibitory neurons play key roles in information processing in neural circuits, despite forming a relatively small minority (∼10%–30% neocortex and <6% in regions of the cerebellar cortex) (Hendry et al., 1987; Korbo et al., 1993). They control the gain and offset of downstream neurons (Mitchell and Silver, 2003; Prescott and De Koninck, 2003), shape neuronal selectivity (Grienberger et al., 2017; Liu et al., 2011; Priebe and Ferster, 2008), and control the precision of spike timing (Duguid et al., 2012; Pouille and Scanziani, 2001; Swadlow, 2003). Studies of individual neurons have shown that different types of inhibitory interneurons exhibit a range of cellular and synaptic properties that support these diverse functional roles (Isaacson and Scanziani, 2011; Kepecs and Fishell, 2014; Tremblay et al., 2016). By contrast, much less is known about the functional properties of interneurons at the population level as their low density has made it difficult to measure population activity with conventional methods. However, recent advances in microscopy and electrophysiological probes have opened up the possibility of studying the properties of local inhibitory circuits (Geiller et al., 2020; Khan et al., 2018; Ma et al., 2020; Najafi et al., 2020).

Golgi cells (GoC), the main inhibitory interneuron in the cerebellar input layer, receive excitation from mossy fibers and granule cells (GrCs) (Cesana et al., 2013; Dieudonné, 1998; Eccles et al., 1966; Kanichay and Silver, 2008). GoCs modulate the threshold and gain of vast numbers of downstream GrCs (Billings et al., 2014; Mitchell and Silver, 2003; Rothman et al., 2009), as well as govern their spike timing (Duguid et al., 2015) through feedforward and feedback inhibition. These functions are thought to conserve information, make sparse and decorrelate GrC activity, and introduce spatiotemporal patterns at the population level, aiding pattern separation (Cayco-Gajic and Silver, 2019) and temporal processing (D’Angelo and De Zeeuw, 2009; Mapelli et al., 2010). Although sensory-evoked responses of individual GoCs (Duguid et al., 2015; Edgley and Lidierth, 1987; Holtzman et al., 2006; van Kan et al., 1993; Vos et al., 2000) have been studied *in vivo*, experimental evidence for how their activity is organized at the population level is lacking. GoCs are electrically coupled together (Dugué et al., 2009) via connexin 36 gap junctions (GJs), formed between the dendrites of neighboring cells (Szoboszlay et al., 2016; Vervaeke et al., 2010, 2012). Computational models of GoC circuits suggest that excitatory synaptic input can either synchronize or desynchronize population activity (Dugué et al., 2009; Maex et al., 2000; Vervaeke et al., 2010). These model-based predictions are consistent with *in vivo* experimental observations of synchrony between pairs of GoCs (Dugué et al., 2009; van Welie et al., 2016; Vos et al., 1999) and local field potential (LFP) recordings showing that coherent oscillatory activity only occurs during immobile states (Hartmann and Bower, 1998), which could reflect differing levels of synchrony in the GoC network. Quantifying GoC population dynamics in awake animals is therefore key for understanding how this electrically coupled inhibitory network can regulate downstream activity on different spatiotemporal scales.

## Results

**Figure 1:**
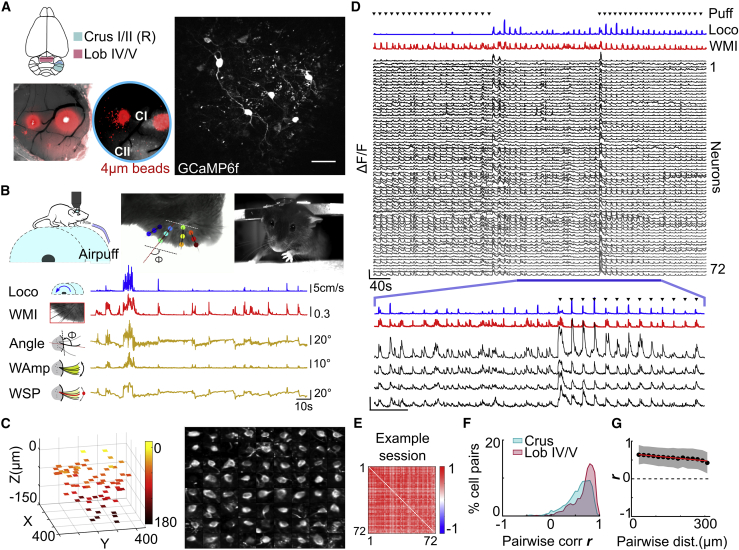
Population imaging of cerebellar Golgi cells (GoCs) (A) Left: schematic of mouse brain with cerebellar regions Crus I/II (cyan) and Lobule IV/V (magenta). Example viral vector injection site with red microbeads and overlaid GCaMP6f fluorescence in white; CI, Crus I; CII, Crus II. Right: example plane with GCaMP6f-expressing GoCs. Scale bar, 50 μm. (B) Top: behavioral setup for 3D 2-photon imaging of head-fixed mouse on cylindrical wheel. Example frames from whisker and front video cameras and offline tracking of multiple single whiskers. Colors indicate 12 different points tracked. Bottom: time course of locomotion (Loco), whisker motion index (WMI), whisker angle (angle), whisking amplitude (WAmp), and whisker set point (WSP) from an example session. (C) Left: regions of interest (ROIs) centered on GoC somata in Crus I/II located within the imaging volume, to be selectively imaged with patch scanning. Color bar indicates depth (μm) from the top of the imaged stack. Right: montage of mean images of the GoC soma from each ROI. (D) ΔF/F traces for a single imaging session, for each of the ROIs in (C), arranged by depth together with the Loco and WMI. Timing of air puff on whiskers (puff), black triangles. Bottom: expanded timescale for 4 example neurons. Scale bars, 100% ΔF/F and 40 s. (E) Pairwise correlation matrix for population activity in (D). (F) Distribution of pairwise correlation *r* (ΔF/F) across all experiments for both cerebellar lobules (n = 7,669 pairs/N = 5 animals for Crus, n = 1,199/N = 4 for Lob IV/V). (G) Relationship between activity correlation (*r*, for ΔF/F) and distance between GoC pairs. Black symbols indicate the binned population mean, shaded area denotes standard deviation (SD), red line indicates linear fit (n = 8,868 pairs/N = 9 animals). See also Figures S1–S3.

To investigate population dynamics in local GoC circuits, we monitored their activity with GCaMP6f (Chen et al., 2013), which was selectively expressed in the majority of cerebellar GoCs in the injected region (Figures 1A and S1). We targeted two cerebellar lobules that differ in the composition of their inputs, phylogenetic history, and function (Apps et al., 2018; Stoodley and Schmahmann, 2010)—Crus I/II in the cerebellar hemisphere, which in rodents primarily receives whisking and other orofacial sensorimotor inputs (Van Ham and Yeo, 1992; Proville et al., 2014; Shambes et al., 1978), and lobule (Lob) IV/V in the cerebellar vermis, which receives inputs from the spinocerebellar tract and is involved in posture and locomotion (Chambers and Sprague, 1955; Luo et al., 2017). Mice were head fixed and placed on a treadmill, where they were free to perform a range of behaviors. We focused on two voluntary motor behaviors, locomotion and whisking, and on responses to an unexpected, brief (100 ms) minimally aversive air puff on ipsilateral whiskers (Figure 1B). Population activity was monitored with an acousto-optic lens (AOL) three-dimensional (3D) 2-photon microscope (Nadella et al., 2016) by rapidly and selectively imaging the GCaMP6f-expressing GoC somata distributed throughout the imaging volume (∼300 × 300 × 150 μm) using small imaging patches (e.g., ∼40 × 20 μm; Figure 1C; Video S1). This random-access approach, which avoided imaging the dead space between sparsely distributed cell bodies, enabled us to record simultaneously from tens of GoCs (mean ± SD = 27 ± 10 cells, range = 10–72 cells, N = 9 animals) at video rates (43 ± 20 Hz, range = 18–97 Hz). This dense sampling of the local inhibitory circuit (Video S2) revealed activity that was widespread across the GoC population (Figure 1D).

Video S1. Acousto-optic lens 3D 2-photon imaging z stack and selection of regions of interest for imaging Golgi cell somata, related to Figure 1 Zstack performed with acousto-optic lens-based remote focusing and scanning showing Golgi cells (GoCs) distributed through the 400 μm × 400 μm × 220 μm imaging volume. Sequence of planes, with 72 “patches” placed to selectively image GoC somata with 3D random-access imaging. Same example as Figure 1D.

Video S2. Acousto-optic lens 3D 2-photon patch imaging of Golgi cell somatic activity in Crus I of an awake, head-fixed mouse (without real-time motion correction), related to Figure 1 Video of 72 simultaneously imaged patches with Golgi cell (GoC) somata expressing GCaMP6f, same example as Figure 1D. Patches (20 μm × 26 μm) are located at different depths within the granule cell layer in right Crus I. Locomotion trace shown below with a moving red bar to indicate locomotion aligned with the imaging time point. Data were acquired without real-time movement correction, but frames were registered post-hoc. The acquisition rate was 18 Hz, with 90 s of playback at 10×.

### Highly correlated GoC activity

Increases in GoC population activity were associated with periods of spontaneous whisking and locomotion, and when mild air puffs were applied to the whiskers (Figures 1D and S2). Pairwise correlations in GoC activity were large and positive (0.58 ± 0.10, n = 21 sessions, N = 9 animals; Figures 1E and 1F), and no differences in average correlations per session were observed between Crus I/II and Lob IV/V (0.57 ± 0.08, n = 16/N = 5 versus 0.63 ± 0.14, n = 5/N = 4, Mann-Whitney *U* test, p = 0.3). Since the high level of correlation could be distorted by the slow decay kinetics of the GCaMP6f indicator (Sabatini, 2019), we performed a complementary analysis using an established spike estimation algorithm (Berens et al., 2018; Deneux et al., 2016) to infer the underlying spike-related events (Figures S3A and S3B). GoC event rates exhibited similar population-level changes associated with whisking and locomotion, and substantial pairwise correlations (Figure S3C; 0.42 ± 0.10, n = 21/N = 9; events versus ΔF/F = −0.16 ± 0.11, p = 1.4 × 10^−^^4^; Wilcoxon signed rank test). Correlations remained high across the imaging volume, with little decay with distance for both ΔF/F (Figure 1G; linear fit: slope = −0.18/300 μm, R^2^ = 0.88) and event rates (Figure S3D**,** linear fit: slope = −0.02/300 μm, R^2^ = 0.08). These results show that local populations of GoCs exhibit spatially uniform, highly correlated activity in awake behaving mice.

### Slow network-wide activity modulation across local networks

**Figure 2:**
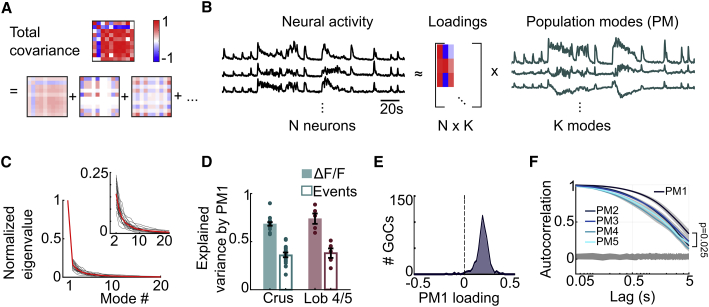
Slow network-wide activation of GoCs (A) Illustration of how correlations between GoCs were quantified by decomposing total covariance into contributions from different modes. (B) Illustration of extraction of population modes from neural activity using principal-component analysis. Time-varying neural activity (left), the loading matrix (center), and activity along population modes (right). The loading matrix gives the weight of mode *k* for neuron *n*. (C) Distribution of eigenvalues for the population covariance matrix (for ΔF/F), normalized by the maximum eigenvalue in each session. Black lines are individual sessions (n = 21 sessions, N = 9 mice), solid red line is the mean across sessions. Inset shows the expanded axis without population mode 1 (PM1). (D) Cross-validated explained variance by first PM alone for ΔF/F (solid) and events (open) in Crus I/II (cyan) and Lob IV/V (magenta). Points indicate mean across neurons on an individual session; shaded bar and error bar indicate mean across sessions and SEM, respectively. (E) Distribution of loadings for top mode (PM1, n = 582 neurons/N = 9). (F) Autocorrelogram for the first 5 population modes (darkest PM1, lightest PM5); shaded areas denote SEM across sessions. Gray area at bottom shows the 95% confidence interval for the time-shuffled control. p value reported at 5-s lag (PM1 versus non-PM1); Wilcoxon signed rank test, n = 21/N = 9. See also Figure S3.

The structure and distribution of correlations is generally more important than their mean strength in determining information encoded by neural populations (Averbeck et al., 2006; Cohen and Kohn, 2011). We therefore examined the correlation structure by decomposing total covariance into contributions from different population modes (PMs, or degrees of freedom; Figure 2A) using principal-component analysis (PCA), which enabled each neuron’s activity to be expressed as a weighted linear combination of the different PMs, with the weights expressed as the loadings (Figure 2B). The contribution of each mode to the total population covariability was calculated using a cross-validated variant of PCA (Owen and Perry, 2009; Stringer et al., 2019). This revealed a dominant first population mode (PM1), with an amplitude (eigenvalue) almost 6 times larger than the next mode (Figure 2C; PM2/PM1 = 0.16 ± 0.05, n = 21/N = 9). PM1 explained a large fraction of single neuron variability (cross-validated explained variance [CVEV] = 0.70 ± 0.09, n = 21/N = 9 for all recordings), and no difference was observed between Crus I/II and Lob IV/V (Figure 2D; Mann-Whitney *U* test, p = 0.48).

To investigate whether PM1 corresponded to a network-wide modulation of GoCs rather than a subset of highly active neurons, we examined the loading of PM1 for individual neurons. Most GoCs in each circuit had a large positive weight for PM1, suggesting that they were all similarly activated in a common mode (Figure 2E). Comparison of the autocorrelation of the top modes during the same behavioral sequences revealed that PM1 had the slowest decay (Figure 2F; PM1 versus non-PM1: Δcorr at 1-s lag = 0.12 ± 0.02, p = 9 × 10^−5^; Δcorr at 5-s lag = 0.15 ± 0.06, p = 0.025; Wilcoxon signed rank test, n = 21/N = 9), suggesting that PM1 arises from a slow modulation of the network activity. To test how the slow time course of GCaMP6f fluorescence affected the relative amplitudes of the PMs, we repeated the analysis on extracted event rates. This confirmed that PM1 was substantially larger than subsequent modes (Figure S3E; PM2/PM1 = 0.21 ± 0.09), with positive weights of PM1 for most neurons (Figures S3F and S3G). However, the amplitude of PM1 was lower for events than for fluorescence (Figure 2D, unfilled bars; CVEV by PM1: 0.36 ± 0.10, n = 21/N = 9; events versus ΔF/F, p = 6 × 10^−5^, Wilcoxon signed rank test), and the autocorrelation timescale faster (Figure S3H; PM1 versus non-PM1: Δcorr at 500-ms lag = 0.20 ± 0.04, p = 1 × 10^−4^; Δcorr at 5-s lag = 0.06 ± 0.03, p = 0.43; Wilcoxon signed rank test, n = 21/N = 9). The qualitatively similar results obtained for events show that the highly correlated GoC activity reflects a slow, network-wide modulation of GoCs present in both the vermis and the cerebellar hemisphere.

### Widespread network activity is correlated with the level of behavioral activity

**Figure 3:**
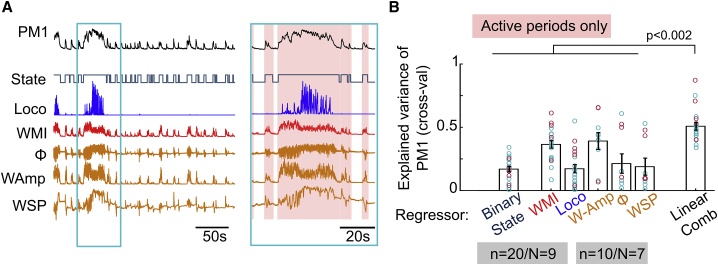
Network-wide modulation of GoCs is correlated with behavioral engagement (A) Example of PM1, binary state and behavioral variables (whisker angle, Φ). Blue box shows the indicated region on expanded timescale, with active periods marked in light red. (B) Cross-validated linear regression of PM1 to all behaviors. Explained variance (mean ± SEM): (state) 0.17 ± 0.03, (WMI) 0.36 ± 0.03, (Loco) 0.17 ± 0.03, (W-Amp) 0.39 ± 0.07, (Φ) 0.21 ± 0.08, and (WSP) 0.19 ± 0.07. Linear combination (Linear Comb, 0.49 ± 0.15) predicted PM1 significantly better than any of the individual behavioral variables (Mann-Whitney *U* test). Number of sessions (n) and animals (N) analyzed. Scatter represents individual sessions (Crus: cyan, Lob IV/V: magenta), bars and error bars indicate means ± SEMs across sessions. See also Figure S4.

To investigate the relationship between the common population mode and behavior, we compared the amplitude of PM1 to multiple behavioral variables: locomotion speed (Loco), whisker motion index (WMI), whisker setpoint (WSP), amplitude (WAmp) and angle (Φ). We also defined a binary variable indicating whether the animal was in an active state (periods of locomotion and whisking) or a quiet wakeful state (Figure 3A). PM1 was correlated with all of the measured behavioral variables (Figures S4A and S4B), which were themselves partially correlated with one another due to coordinated body movements (Figure S4C). The level of correlation of PM1 with behavior was similar for Crus I/II and Lob IV/V, suggesting that it reflects widespread GoC activation during active behavioral states.

To test whether specific behaviors were encoded in the common mode, we examined only the active state (including 500 ms before and after each active epoch; see box in Figure 3A) using linear regression. A modest fraction of the PM1 variance could be explained by each of the measured behavioral variables (Figure 3B; ∼30%–40% for WMI and W-Amp, 17%–20% for the others), and their contributions were similar across lobules (Crus I/II versus Lob IV/V: (WMI) p = 0.08, (Loco) p = 0.08, Mann-Whitney *U* test). A linear combination of behavioral measures accounted for a substantially larger fraction of the common mode variance than the individual behavioral variables (Figure 3B; ∼40%–60%, p < 10^−4^ for WMI, Loco, n = 20 sessions/N = 9 animals; p < 0.001 for WAmp, angle Φ, and WSP, n = 10/N = 7; one-sided Wilcoxon signed rank test), with cross-validation ensuring a fair comparison of models with differing numbers of parameters. Moreover, the binary state variable explained less variance than whisking amplitude (WAmp versus state: p = 0.003, n = 10/N = 7, Wilcoxon signed rank test). Thus, the widespread engagement of the GoC network, as reflected in PM1, is correlated with the overall level of whisking and locomotion, rather than distinct behavioral variables in different regions of the cerebellar cortex.

### GoCs exhibit heterogeneous response dynamics

**Figure 4:**
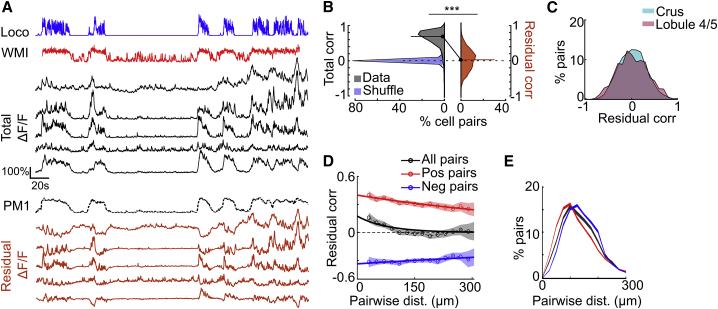
Spatially mixed heterogeneous dynamics within local GoC networks (A) Example activity traces from 5 GoCs (ΔF/F, black traces) during whisking (WMI, red) and locomotion (Loco, blue), with different activity during active states as residual ΔF/F (orange) after projecting out PM1. Mean total correlation = 0.72, residual correlation = 0.07. (B) Comparison of pairwise correlations for total (gray) and residual (orange) ΔF/F, and for shuffle control (light blue) across sessions (n = 21, N = 9 animals). Black symbols and horizontal bars show population means. Difference in total and residual correlation for all pairs, p = 6 × 10^-5^, Wilcoxson signed-rank test. (C) Distribution of residual correlations (after projecting out PM1) in Crus I/II (cyan, n = 16, N = 5) and Lob IV/V (magenta, n = 5, N = 4). (D) Dependence of residual correlation on distance between GoCs, for positively (red; Pos) and negatively (blue; Neg) correlated pairs (significant compared to each pair’s shuffle distribution) and all pairs (black). Dotted lines with circles represent binned averages (20 μm bins), shaded areas indicate SEMs, and solid lines denote exponential fits to data. (E) Distribution of distance between GoCs for positively (red) and negatively (blue) correlated pairs and all pairs (black), for 20 bootstrapped samples. Shaded region shows means ± SEMs. See also Figures S3 and S5.

We next examined whether the slow common modulation of the network implied homogeneous activity across individual neurons or whether they also exhibited distinct dynamics on subsecond timescales. Visual inspection of ΔF/F from different GoCs in the same circuit indicates that they have heterogeneous activity profiles during periods of active whisking and running (Figure 4A). To quantify the variability of individual neurons around the slow common population mode, we projected out PM1 and examined their residual ΔF/F (Figure 4A). Without this procedure, the total correlations were largely positive due to the large amplitude of PM1, which masked the residual correlations (Figures 4B and S5A–S5C; total versus residual correlations: [mean ± SD] Δcorr = 0.57 ± 0.09, n = 21/N = 9, p = 6 × 10^−^^5^, Wilcoxon signed rank test). Residual correlations were distributed around zero (mean = 0.01 ± 0.03, n = 21/N = 9), but included strongly positively and negatively correlated GoC pairs (significant compared to shuffled control for each pair; Method details) with an absolute magnitude of significant correlations (0.32 ± 0.16). Residual correlations were similar for both lobules (Figure 4C; Crus I/II versus Lob IV/V, p = 0.6, Mann-Whitney *U* test), and were robust when calculated using event rates rather than ΔF/F (Figures S3I and S3J; residual correlation = −0.01 ± 0.01, absolute magnitude = 0.18 ± 0.11, total versus residual correlation, Δcorr = 0.42 ± 0.10, p = 6 × 10^−5^, Wilcoxon signed rank test). Although residual correlations decayed with distance (Figure 4D, exponential fit in 20-μm bins: all, λ = 37 μm, R^2^ = 0.85, n = 21/N = 9), the separation of positively and negatively correlated pairs showed that they exhibited much weaker distance dependencies (positively correlated, λ = 625 μm, R^2^ = 0.92; negatively correlated, λ = 1,250 μm, R^2^ = 0.68), with negatively correlated pairs found at slightly larger distances (Figure 4E; Kolmogorov-Smirnov test, p < 10^−4^). We also examined the spatial dependence in residual correlations when aligned along, or orthogonal to, the parallel fiber axis, as this should reveal any on-beam versus off-beam spatial structure. However, comparably weak spatial dependencies were observed in these two directions (Figure S3K). These results suggest that despite the slow shared modulation of the network activity during spontaneous behaviors, groups of individual GoCs dispersed throughout the local circuit also exhibit distinct dynamics on faster timescales.

**Figure 5:**
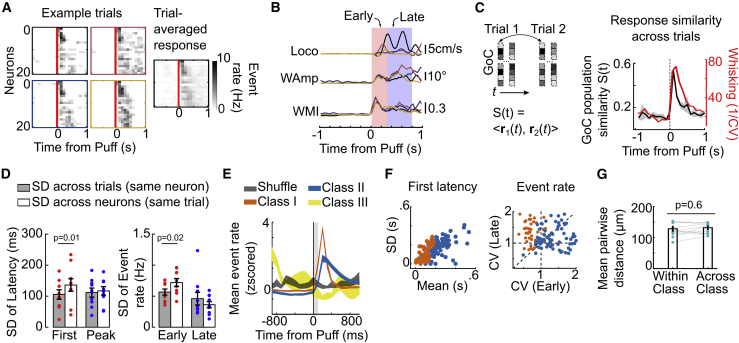
Reliable heterogeneous responses of local GoC network to air puff to the whisker (A) Event rates for 20 GoCs on 4 individual trials, and trial-averaged responses aligned to air puff applied to the ipsilateral whiskers (red bar) for an example session. (B) Air puff-triggered Loco, WAmp, and WMI for the same example sessions as in (A). Early (0–300 ms) and late (300–800 ms) epochs, characterized by low and high behavioral variability, respectively. (C) Left: schematic showing similarity of GoC response *S*(*t*); dot product between population activity vectors *r*(t) for same time bins *t* aligned to puff for each pair of trials, and averaged across all pairs. Right: profile of similarity across trials for GoC population response (black) and WMI (red), as a function of time from puff onset. CV, coefficient of variation. Shaded region (gray) shows SEM across sessions (n = 12/N = 5 animals). The same sessions were used in (C)–(G). (D) Inter-trial (filled) and intra-trial (white) variability in response to air puff. Left: SD of latency to first event and peak event rate, for single neurons across trials and across neurons within the same network. Right: inter-trial and intra-trial SD of event rate for early (0–300 ms; red) and late (300–800 ms; blue) periods. Scatter denotes individual session; bar and error bar denote means ± SEMs across sessions. (E) Mean air puff-triggered response (event rate, with PM1, *Z* scored for each type) for 3 classified types (n = 85,161,15 GoCs in classes I, II, and III, respectively, N = 5 animals), compared to time-shuffled responses (gray). (F) Response variability for class I and II GoCs. Left: SD and mean latency to first event across trials for all class I (orange) and class II (blue) GoCs. Right: CV for event rate during early and late epochs (n = 261 neurons). (G) Mean distance for GoCs based on puff response (grouped by class in E). No significant difference between within-class and across-class distances. Scatter represents individual sessions; bars with error bars represent means ± SEMs across sessions. See also Figure S6.

To investigate the reliability of GoC responses in Crus I/II, we quantified responses to brief air puff stimuli to the whiskers (events in 50-ms bins; Figures 5A and S6A), which triggered stereotyped whisker deflection at early times followed by variable self-generated whisking and locomotion (Figure 5B). To determine the reliability of population activity, we computed the similarity of air puff-triggered GoC population activity vectors across trials, at different times from puff onset (Figure 5C). Comparison of the similarity in GoC population activity with the trial-to-trial similarity in WMI revealed that GoC network activity and whisker motion is highly stereotyped right after puff, and that their trial-to-trial similarity decays back to baseline level on a comparable time course (weighted decays, 460 and 530 ms, respectively). Correlations between GoCs within local networks were, however, broadly distributed (Figure S6B), suggesting heterogeneity of individual GoC responses. Air puff-triggered responses of individual GoCs had a mean first latency of 103 ± 133 ms with substantial inter-trial variation in onset (SD of latency to first event = 108 ± 99 ms) and event rate (SD in early epoch [0–300 ms], across-trial = 0.57 ± 0.15Hz; Figures 5A and 5D). However, the response variability across neighboring GoCs within a single trial was consistently larger than for a given GoC across trials (SD of latency to first event = 136 ± 60 ms, p = 0.01 and the SD of early event rate = 0.72 ± 0.19 Hz, p = 0.02; Wilcoxon signed rank test for inter-trial versus intra-trial SD; n = 12/N = 5; Figure 5D). After the first 300 ms, the trial-to-trial variability was comparable to that across the local population (SD of response in late epoch, across-trial = 0.46 ± 0.31Hz, across-neurons = 0.36 ± 0.15 Hz, p = 0.08), consistent with behavioral variability across trials in the late epoch (Figures 5B and 5C). We further classified GoCs into 3 broad categories: fast onset and transient response (class I, 85/261 cells; 34% ± 8% per session), late onset and/or sustained activity (class II, 161/261 cells, 61% ± 8% per session), and depression or no response (class III, 15/261 cells; Figures 5E and S6). Class I responses were highly reliable, with ∼90% response probability and low trial-to-trial variability in first latency (Figures S6E and S6F). By contrast, class II had higher intertrial variability, consistent with the larger variability of self-generated behavioral responses at later times (Figures 5F and S6F). There was no difference in pairwise distance between GoCs within or across these classes, indicating that they were spatially mixed within the local circuit (Figure 5G; Wilcoxon signed rank test, p = 0.64). The larger within-network variability than inter-trial variability reveals that GoC circuits respond reliably to mild air puffs to the whiskers and that individual GoCs within local populations exhibit heterogeneous dynamics on the timescale of hundreds of milliseconds.

### Differential modes in population activity

**Figure 6:**
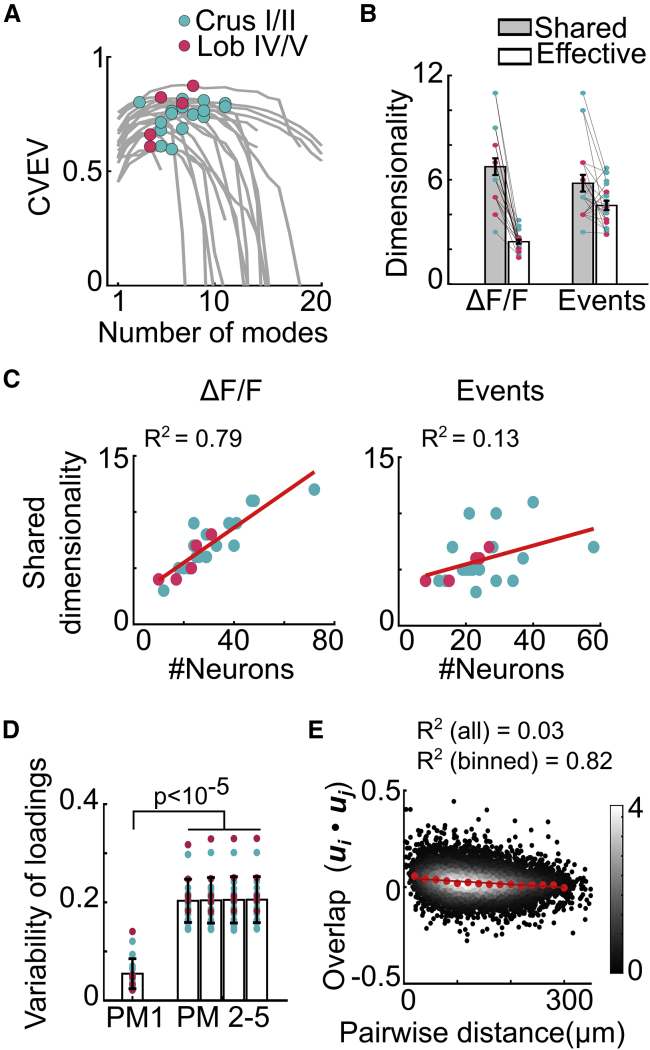
Multidimensional population activity (A) Relationship between cross-validated explained variance (CVEV) and number of population modes (PMs). Gray curves show individual sessions and filled circles show peak CVEV, corresponding to shared dimensionality for populations in Crus I/II (cyan, n = 16/N = 5) and Lob IV/V (magenta, n = 5/N = 4). Same sessions and color scheme were used in (B)–(F). (B) Dimensionality of GoC responses quantified either as shared or effective dimensionality. (C) Estimated shared dimensionality from recordings with different numbers of GoCs for ΔF/F (left) and event rates (right). Red lines denote linear fits. (D) Variability (SD) of loadings along top PMs (PM 1–5) within each session. (E) Similarity between GoCs as measured by overlap (dot product) of their loadings as a function of their pairwise distance. Red symbols indicate binned averages and line denotes linear fit. Color bar indicates probability density. For all of the panels, scatter denotes individual sessions; bars and error bars indicate means ± SEMs across sessions. See also Figure S7.

To investigate how population activity is structured across GoCs, we quantified the shared activity subspace, using the peak of the relationship between CVEV and the number of modes (Owen and Perry, 2009; Stringer et al., 2019) (Figure 6A). This provided a lower bound on the dimensionality of the shared subspace (shared dimensionality) that can be inferred from the population activity, given the noise level within each experiment and the size of the imaged population. Similar estimates of shared dimensionality were obtained for GoC circuits in Crus I/II and Lob IV/V (p = 0.18, Mann-Whitney *U* test), and were comparable when calculated from ΔF/F (7.0 ± 2.4) or event rates (5.9 ± 2.2, n = 21/N = 9; Figure 6B). However, these are likely to be underestimates for the entire local network, as higher values were associated with experiments with larger numbers of recorded cells (Figure 6C; linear fit for shared dimensionality versus population size [ΔF/F] R^2^ = 0.78, [events] R^2^ = 0.13).

An alternative approach that characterizes the dimensionality of total variability (including independent GoC activity and noise) after adjusting for the relative amplitudes along the different modes to give an effective dimensionality (Abbott et al., 2011) gave a lower estimate due to the large, possibly overestimated, contribution of PM1 (Method details; Figure 6B; effective dimensionality: ΔF/F = 2.5 ± 0.5, events = 4.5 ± 1.2; shared versus effective: ΔF/F p = 4 × 10^−5^, events p = 0.01, Wilcoxon signed rank test). Despite the differences in these approaches, these results revealed that local GoC circuits exhibit multidimensional population activity, with some response dynamics shared across the entire population and others restricted to subsets of GoCs.

To examine the properties of population modes beyond PM1, we compared their loadings to the common mode within each population. Their substantially higher variance indicates that they are differentially distributed across the GoC population (Figure 6D; PM1 versus PM2–PM5, p < 10^−5^, Wilcoxon signed rank test). Since these modes contribute to the heterogeneous (Figures 4 and S5) and faster-varying (Figure 2F) residual activity, we refer to them as differential modes. To investigate the organization of the activity space, without the constraint of orthogonality imposed by PCA, we applied independent-component analysis (ICA). ICA gave qualitatively similar results for the dimensionality and the variance of the loading as cross-validated PCA (Figure S7). The angle between PM1 and the differential population modes was close to orthogonal (84° ± 6°, n = 21/N = 9). This suggests that the common and differential population modes are likely to operate independently without interfering with each other.

To explore the spatial structure of GoC responses along differential population modes and to test whether nearby neurons had similar residual modulation, we calculated the dot product of the loading vectors for each pair of neurons (excluding loading of PM1 and noise modes; see Method details) and plotted this as a function of their pairwise distance. The lack of spatial dependence in the overlap of differential modes (Figure 6E; linear fit, slope = −0.05/300 μm, R^2^ = 0.82; Figure S7E for ICA) suggests that differential population modes were neither global nor clustered, which is consistent with the weak distance dependence of residual correlations (Figure 4D).

**Figure 7:**
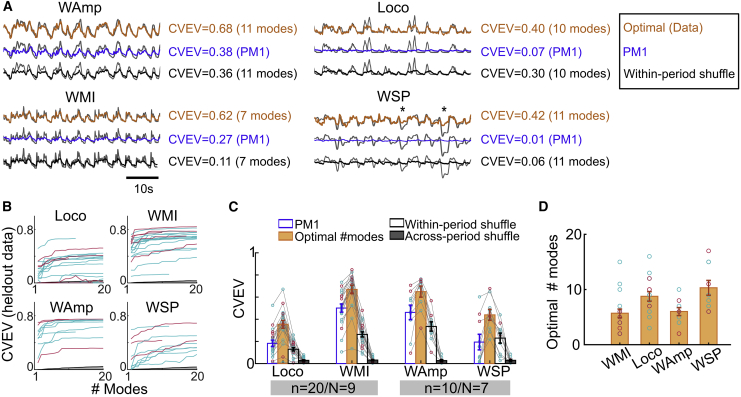
Decoding behavioral variables from GoC population modes (A) Example decoding of 4 behavioral variables (on the same held out time period), using only PM1 (blue) or an optimal number of modes (orange), and for within-period shuffle control (black), with the corresponding CVEV for each case. The behavioral trace is displayed in gray. (B) CVEV for increasing number of modes for 4 behavioral variables for all sessions (n = 20 for WMI, Loco; n = 10 for WAmp, WSP; cyan for Crus I/II, magenta for Lob IV/V). Same sessions are shown in (C) and (D). (C) CVEV by decoding individual behavioral variables from PM1 (blue, unfilled) or an optimal combination of modes including PM1 (orange, filled). Shuffle controls (black): within-period shuffle (unfilled) in which the 500-ms blocks from the same active period were rearranged, and across different active periods (filled) in which 500-ms blocks were randomly reordered across all active periods. (D) Number of modes for optimal decoding performance (90% of the total improvement over PM1) for each of the 4 behavioral variables. See also Figure S8.

To investigate whether the differential population modes contain sensorimotor information, we used linear regression to decode behavioral variables from one or more population modes (Figures 7A and 7B). An optimal number of population modes (including PM1) explained a larger fraction of the CVEV for each of the behavioral parameters than the common mode alone (Figure 7C, PM1 versus optimal modes: Loco, ΔfEV = 0.16 ± 0.11, p = 10^−4^; WMI, ΔfEV = 0.17 ± 0.06, p = 10^−4^ (n = 20/N = 9) W-Amp, ΔfEV = 0.19 ± 0.13, p = 0.002; WSP, ΔfEV = 0.25 ± 0.12 p = 0.002 (n = 10/N = 7); Wilcoxon signed rank test). The number of optimal modes varied considerably across animals (Figures 7B and 7D; WMI, mean = 5.6 ± 3.2; Loco, 8.7 ± 3.3, n = 20/N = 9; WAmp, 6.0 ± 2.2; WSP, 10.3 ± 4, n = 10/N = 7), likely due to inter-animal differences in behavioral sequences (behavioral information was not directly used for decomposing population activity into modes). However, the consistent increase in explained variance over the common mode indicates that successive differential modes carry additional behavioral information. To confirm this, we performed a control by testing the decodability of 2 shuffled surrogate variables with an optimal number of modes. First, we shuffled traces (as 500 ms blocks) within the same behavioral epoch, thereby maintaining the average level of behavioral activity but removing within-epoch signal structure. As expected, the optimal within-period shuffling performance was similar to the decoding of behavior from PM1 alone (Figure 7C; PM1 versus within-period shuffling: Loco, p = 0.10; WMI, p = 10^−4^; W-Amp, p = 0.04; WSP, p = 0.70; Wilcoxon signed rank test), but was significantly worse than the optimal decoding of true behavioral traces (Figure 7C; optimal mode versus within-period shuffling: Loco, p = 0.001; WMI, p = 10^−4^; W-Amp, p = 0.002; WSP, p = 0.002; Wilcoxon signed rank test). Second, shuffling behaviors across different active periods disrupted the slow dynamics and destroyed all explanatory power (Figure 7C; PM1 versus across-period shuffle: Loco, p = 2 × 10^−4^; WMI, p = 10^−4^; W-Amp, p = 0.002; WSP, p = 0.10; Wilcoxon signed rank test). In a separate set of experiments with high-speed tracking of the ipsilateral forelimb, we observed that some GoCs (24%) were more active during certain phases within each step cycle (Figure S8). This suggests that the rapidly modulated activity of subsets of GoCs encode features such as paw lift during the step cycle. These results show that differential GoC activity contains specific information on whisking and locomotion.

### An electrically coupled GoC circuit model reproduces observed population-level properties

**Figure 8:**
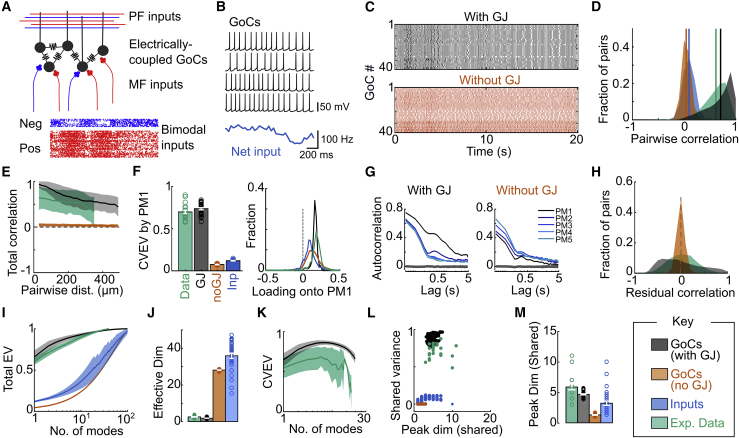
Biologically detailed GoC circuit model requires electrical coupling to reproduce multidimensional network activity with a common mode (A) Schematic of GoC circuit model, with gap junctions (GJs, resistor symbols) between cells and positively (Pos, red) and negatively (Neg, blue) modulated input spike trains targeted to excitatory synapses on apical dendrites (PF, parallel fiber inputs) and soma (MF, mossy fiber inputs) of model GoCs. (B) Example of somatic membrane potentials from model GoCs and net input rate. (C) Raster plots of spiking activity from 40 GoCs with GJs (top, black) and without GJs (orange, bottom). (D) Distribution of pairwise correlations between GoCs within local circuits with electrical coupling (black) and without electrical coupling (orange) together with correlations between inputs (blue) across varying input levels. The distribution of correlations observed experimentally with *in vivo* calcium imaging is shown in green. (E) Dependence of total pairwise correlation on distance between model GoC somata and in experimental data. (F) CVEV by PM1 (left) and (right) distribution of loadings for PM1, as measured experimentally (data), and for model circuits with (GJ) and without (noGJ) electrical coupling, together with PM1 of the simulated inputs (Inp). (G) Autocorrelation of PM1–5 for modeled GoCs with (left) and without (right) GJs, as average profile across simulations with different input levels. The gray shaded area indicates autocorrelation of shuffled activity. (H) Distribution of residual correlations (after projecting out PM1) across all input levels. (I) Relationship between total explained variance (EV) and number of modes. (J) Effective dimensionality (based on I) of population activity of GoCs and inputs. (K) Relationship between CVEV and number of modes, for experimental data (green) and modeled GoCs with GJs (black). Bold lines are means across sessions and shaded regions SDs. (L) Maximal CVEV (shared variance) versus number of modes at peak CVEV (shared dimensionality). (M) Dimensionality of shared subspace: number of modes corresponding to maximal CVEV for each simulation or experimental session. (D–M) Key for color labels. For all relevant panels, scatter denotes different network simulations (with a given input level) or experimental sessions. See also Figures S9 and S10.

To investigate how population modes may arise, we built a biologically detailed model of the GoC circuit that was constrained by experimental measurements (Figure 8A; Method details). Model GoCs reproduced the narrow spike and slow afterhyperpolarization (AHP) profiles (Figure 8B), spontaneous firing rate and firing rate-current relationships recorded from GoCs (Lanore et al., 2019). A total of 115 model GoCs were randomly placed within a 500 × 500 × 100 μm volume and connected via multiple linear GJ conductances, such that the coupling coefficient and connection probability between pairs of GoCs decayed with space constants of 70 and 40 μm, respectively, as measured in paired whole-cell recordings (Vervaeke et al., 2010). Excitatory mossy fiber and GrC synapses were randomly connected to somata and dendrites, respectively, and input trains mimicked positively and negatively modulated mossy fibers (Roš et al., Cosyne abstract 2018) and parallel fibers (Lanore et al., 2021) observed during spontaneous behaviors. Groups of model GoCs (35–45) located within subregions equal in size to our imaging volumes (300 × 300 × 100 μm) were analyzed to facilitate comparison with our experimental results.

Spike trains from model GoCs were loosely synchronized across the local population (Figure 8C). GoCs exhibited strong positive pairwise correlations (Figure 8D, mean correlation = 0.64 ± 0.27) that were robust across a wide range of excitatory drive (Figure S9). Moreover, there was little decay in the correlation magnitude across the imaging volume, as observed experimentally (Figure 8E), but this became more pronounced on larger spatial scales and when coupling strength or input correlations were reduced (Figures S9 and S10A). Removal of electrical coupling desynchronized GoC spiking and reduced the mean pairwise correlation to zero, close to that of the synaptic inputs (Figures 8C–8E). These results show that electrical coupling could underlie the high pairwise correlations observed across local populations of GoCs during spontaneous behaviors.

To investigate the relationship between electrical coupling and the structure of population activity in the GoC circuit model, we applied PCA. This revealed a slow, widespread, and dominant common population mode (PM1, CVEV = 0.67 ± 0.12), as found experimentally (Figures 8F and 8G), despite a weak PM1 in input population (CVEV = 0.11 ± 0.01). Moreover, projecting out PM1 from the GoC population activity revealed residual correlations in activity with a wide distribution (Figure 8H), indicating the presence of differential modes. This partitioning of total variability between a dominant common mode and few differential modes was similar for both model and experiment (Figure 8I) and was captured by their low effective dimensionality (Figure 8J; model mean = 1.9 ± 0.4, ΔF/F data = 2.5). Remarkably, the elimination of GJs from the model reduced the CVEV of PM1 (Figure 8F) and increased the effective dimensionality to a level that is comparable to the input trains (28 ± 3 and 35 ± 8, respectively), consistent with a role of electrical coupling in quenching independent variability. The effective dimensionality of GoC population variability depended on coupling strength (Figure S10D). To quantify the number of reliable population modes in the model, we examined the relationship between the CVEV and the number of modes (Figure 8K). This revealed a shared dimensionality comparable to that found experimentally (5–6), but the explained variance was larger, which could be due to the lack of noise in the model (Figures 8L and 8M). By contrast, both the synaptic inputs and GoC circuit models lacking electrical coupling exhibited negligible shared variance and low shared dimensionality, due to their activity being largely independent. These results show that the physiological level of electrical coupling between GoCs could quench independent input variability and amplify shared variability of GoCs, generating a dominant common population mode across the entire local GoC population, while also supporting several differential population modes that are restricted to subsets of GoCs.

## Discussion

To investigate population dynamics in an inhibitory circuit, we have imaged GoCs within the cerebellar input layer during whisking and locomotion. On slow timescales (>1 s), population activity was dominated by a network-wide modulation, which correlated with the overall level of locomotion and whisking in both vermal and hemispheric regions of the cerebellar cortex. On faster timescales (<1 s), more specific information on whisking and locomotion was encoded at the population level, across GoCs with heterogeneous response properties. Many of the properties of GoC population dynamics were reproduced in a biologically detailed circuit model when GoCs were electrically coupled via GJs, indicating that they could play a major role in generating multidimensional population dynamics with both widespread and distributed components. These properties of the GoC population activity are well suited to delivering the inhibition required for modulating the threshold and gain of downstream cerebellar GrCs (Billings et al., 2014; Hamann et al., 2002; Mitchell and Silver, 2003; Rothman et al., 2009) and for introducing spatiotemporal patterning (D’Angelo and De Zeeuw, 2009; Duguid et al., 2015; Kanichay and Silver, 2008; Mapelli et al., 2010).

### Experimental approach and analysis

We combined random-access AOL 3D 2-photon microscopy (Nadella et al., 2016) with dimensionality reduction and decoding approaches (Cunningham and Yu, 2014) to examine the properties of GoC population dynamics, which are inaccessible with single-neuron or trial-averaged responses (Arandia-Romero et al., 2017; Averbeck et al., 2006). Since measures of the dimensionality of population activity can be distorted by noise (Stringer et al., 2019) and the slow kinetics of calcium indicators (Sabatini, 2019), we separately examined the shared subspace using a cross-validated variant of PCA and the total variability (using effective dimensionality), and we used inferred spike-related events to partially compensate for the effects of GCaMP6f kinetics. We also confirmed the existence of common and differential population modes using ICA and observed that these modes were orthogonal despite not imposing such a constraint. Although it is likely that we have not captured the full complexity of population activity on timescales <100 ms, due to limitations in the number of cells and the temporal resolution of GCaMP6f, our approach establishes that a local GoC circuit can support multidimensional activity that conveys distinct sensorimotor information, which is orthogonal to a slow network-wide modulation.

### Properties and potential origins of the common population mode

The most striking feature of GoC population activity is the slow, circuit-wide upmodulation when the animal is behaviorally active. This common mode conveys nonspecific information on the intensity of whisking and locomotion, and is present in both Crus I/II and Lob IV/V, which have distinct synaptic inputs and functional specializations (Apps et al., 2018). This observation raises the possibility that the common mode could extend across much of the cerebellar cortex. The presence of a dominant common population mode in our GoC circuit model when it was driven with inputs that were only weakly correlated suggests that it is an emergent property of this inhibitory interneuron circuit. Moreover, the dependence of the common mode on GJs between GoC indicates that electrical coupling is critical for its generation. However, the relative contributions of electrical coupling and input correlations to PM1 remain to be determined. By linking electrical coupling to population modes, our results extend previous *in vitro* studies that suggested excitatory drive via feedforward mossy fiber inputs and feedback excitation from GrCs (Cesana et al., 2013; Huang et al., 2013; Kanichay and Silver, 2008) is shared locally through the dendritic GJs present between GoCs (Vervaeke et al., 2012). While our results provide evidence that electrical coupling between GoCs could quench independent input variability and generate the common population mode, other mechanisms could also contribute. These mechanisms include the slow neuromodulatory mechanisms that encode arousal (Reimer et al., 2016), such as serotonin and norepinephrine, which depolarize and hyperpolarize GoCs, respectively (Fleming and Hull, 2019; Lanore et al., 2019). The development of better tools for acute silencing of GJs beyond pharmacological approaches (which have non-specific effects) and improved fluorescent sensors for neuromodulators (Sabatini and Tian, 2020) would enable experimental testing of these hypotheses. Nevertheless, our current results establish that information on the level of locomotion and whisking, which could serve as a shared contextual signal for coordinating different behaviors, is available to local GoC circuits and predict that electrical coupling plays a key role in its emergence.

### Properties and potential origins of differential population modes

Despite substantial shared modulation within a local circuit, individual GoCs exhibited heterogeneous activity during sustained whisking or locomotion. These heterogeneous GoC responses were spatially mixed and tended to be faster than the population-wide increases, resulting in positive and negative modulations in activity about the common mode, and reflected reliable, distributed sensorimotor information present in local GoC networks. This finding is consistent with LFP recordings from the GrC layer that show that theta oscillations present during quiet wakefulness (Dugué et al., 2009; Hartmann and Bower, 1998) disappear during motor activity, since this suggests the network becomes desynchronized. However, the mechanisms that underlie response heterogeneity and differential population modes are less clear. Temporally dispersed excitatory inputs could provide differential synaptic drive to neighboring GoCs, while the presence of common synaptic input and shared synaptic charge through GJs (Vervaeke et al., 2012) could make their activity more similar. Thus, the strength of electrical coupling appears to be a key factor in determining the freedom that individual GoCs have to respond to distinct synaptic inputs. Our GoC circuit modeling suggests that although electrical coupling at physiologically measured levels generates a common mode, it can also support GoC response heterogeneity with multidimensional population activity. Several factors could contribute to GoC response heterogeneity, including differences in synaptic input strength and membrane excitability, mGluR2 signaling (Watanabe and Nakanishi, 2003), sparse inhibitory synapses formed between GoCs (Hull and Regehr, 2012), and spike phase delays generated by the propagation of inhibitory AHPs through heterogeneous electrical coupling (Vervaeke et al., 2010). We observed a diversity in local GoC response dynamics to mild air puffs, but we did not detect global pauses in inhibitory population activity as reported in anesthetized animals (Duguid et al., 2015; Holtzman et al., 2006; Vos et al., 2000). The absence of long-lasting unresponsiveness in our recordings could be due to additional excitatory inputs from both arousal and air puff-triggered startle responses in the awake state. While the underlying mechanisms of differential modes remain somewhat speculative, our results establish that specific behavioral information is represented by differential GoC activity on subsecond timescales and that this is spatially mixed within each local inhibitory circuit.

### Implications for granule cell inhibition and cerebellar function

Our modeling suggests that GJs between GoCs quench input variability, restructuring higher-dimensional excitatory input (such as that from granule cells; (Lanore et al., 2021)) into lower-dimensional activity that shapes inhibition in the GrC layer. GrC inhibition is mediated by multiple mechanisms that operate on distinct timescales—fast quantal release of GABA onto synaptic GABA_A_ receptors, slower spillover of GABA onto high-affinity extrasynaptic receptors, and tonic activation of extrasynaptic receptors by ambient GABA (Brickley et al., 1996; Crowley et al., 2009; Rossi et al., 2003). The widespread nature of the common GoC population mode, further augmented by GABA spillover and tonic inhibition on slow timescales, is likely to provide behavioral activity-dependent inhibition across local populations of GrCs. Such homogeneous inhibition is a common feature of cerebellar circuit models (Billings et al., 2014; Cayco-Gajic et al., 2017), since it is an effective mechanism for network activity-dependent scaling of both firing thresholds and gain of the downstream GrC population (Mitchell and Silver, 2003; Rothman et al., 2009). At the population level, the role of widespread inhibitory modulation may be to dynamically adjust GrC excitability to a level that ensures nonlinear thresholding for effective decorrelation (de la Rocha et al., 2007), while conserving information transmission through the circuit, under widely different levels of excitatory input (Billings et al., 2014). Optimizing these functions when a substantial fraction of GrCs are active, as found experimentally (Giovannucci et al., 2017), is important for supporting high-dimensional representations in the GrC layer (Cayco-Gajic et al., 2017; Litwin-Kumar et al., 2017), which facilitate pattern separation, a major proposed function of the GrC layer (Albus, 1971; Cayco-Gajic and Silver, 2019; Marr, 1969).

In contrast to the slow common mode, differential population modes are likely to generate distinct spatiotemporal patterns of inhibition onto GrCs on intermediate timescales. Such patterned GoC activity is expected to deliver precisely timed inhibition onto individual GrCs (Duguid et al., 2015; Kanichay and Silver, 2008) and modulate spatial patterns of activity onto the postsynaptic targets of GrCs (Valera et al., 2016). Across the GrC population, asynchronous inhibition generated by heterogeneous GoC responses could enhance temporal diversity in GrCs (Medina and Mauk, 2000), providing the basis for liquid state computations and adaptive filters (Fujita, 1982; Rössert et al., 2015; Tokuda et al., 2021) and sensory prediction (Kennedy et al., 2014; Medina et al., 2000). Lastly, computational modeling of circuits with large feedforward expansion has highlighted the importance of having heterogeneous inhibition for decorrelation (Litwin-Kumar et al., 2017; Zavitz et al., 2020). By establishing the presence of common and differential population modes, our results show that a local Golgi cell circuit exhibits multidimensional population activity with the spatiotemporal properties required for both the temporal processing and pattern separation roles proposed for GrC layer, bridging the gap between these 2 distinct conceptual frameworks.

### Implications for other inhibitory circuits

Here, we leveraged 3D random-access AOL microscopy to densely sample sparsely distributed GoCs and the framework of dimensionality to show that a single cerebellar inhibitory circuit can generate coordinated multimodal activity across multiple spatiotemporal scales. The highly correlated pairwise activity, heterogeneity in response properties, and electrical coupling found in GoCs have also been reported in other inhibitory interneurons (Gaffield and Christie, 2017; Khan et al., 2018; Pinto and Dan, 2015), raising the possibility that they could have similarly organized population dynamics. Recent recordings from other circuits show that task-relevant information is also available in local inhibitory networks (Ma et al., 2020; Najafi et al., 2020). Moreover, our biologically detailed model of the GoC circuit predicts that electrical coupling plays a key role in supporting both common and differential modes. By setting the degrees of freedom of each interneuron, the strength of electrical coupling could provide a powerful mechanism by which to tune interneuron population dynamics and thus the spatiotemporal properties of downstream inhibition. Where interneurons form distinct classes, different modes of inhibitory control could be segregated across interneuron populations with specialized connectivity (Bos et al., 2020; Kepecs and Fishell, 2014) and provide greater control of the neuronal input-output functions via dendritic inhibition (Gidon and Segev, 2012; Lovett-Barron et al., 2012). Population-level recordings will be critical for understanding how different inhibitory interneurons orchestrate the activity of the circuits in which they are reciprocally interconnected.

## STAR★Methods

### Key resources table

**Table:** 

REAGENT or RESOURCE	SOURCE	IDENTIFIER
**Experimental models: organisms/strains**		

GlyT2-Cre transgenic mice	Donated by László Acsády	N/A
GlyT2-GFP transgenic mice	Donated by Zoltan Nusser	N/A

**Bacterial and virus strains**		

AAV9.CAG.flex.GCaMP6f.WPRE.SV40	Addgene	Cat # 100835-AAV9; RRID:Addgene_100837

**Chemicals, peptides, and recombinant proteins**		

Red fluorescent beads	Thermofisher	Cat # F8858
Rabbit Anti-Neurogranin primary antibody (1:500)	EMD Millipore - AB5620	RRID:AB_91937
Goat Anti-Rabbit IgG - Alexa 568 (1:500)	Abcam - ab175471	RRID:AB_2576207
Normal Goat Serum	Sigma-Aldrich	NS02L

**Deposited data**		

Raw and analyzed data	This paper	https://doi.org/10.5522/04/14364845

**Software and algorithms**		

Microscopy software	Nadella et al., 2016	https://github.com/SilverLabUCL/SilverLab-Microscope-Software
Custom data-analysis scripts	This paper	https://github.com/harshagurnani/GoCPopImaging
Computational modeling and analysis	This paper	https://github.com/harshagurnani/GoC_Network_Sim_BehInputs
Custom scripts for preprocessing raw acquisition data	This paper	https://github.com/harshagurnani/CaDataAnalysis_GoC
DeepLabCut: Markerless tracking and pose estimation of animals	Mathis et al., 2018	https://github.com/DeepLabCut/DeepLabCut
MLSpike: Spike-event extraction from calcium imaging data	Deneux et al., 2016	https://github.com/MLspike/spikes
ImageJ (Fiji), Image processing software	Schindelin et al., 2012	https://imagej.net/Fiji

### Resource availability

#### Lead contact

Further information and requests for resources and reagents should be directed to and will be fulfilled by the Lead Contact, Angus Silver (a.silver@ucl.ac.uk).

#### Materials availability

This study did not generate new unique reagents.

#### Data and code availability

The accession number for the preprocessed data reported in this paper is Figshare: https://doi.org/10.5522/04/14364845, and the raw microscopy and video data will be available on request. Scripts for preprocessing 3D imaging and behavioral data are available at https://github.com/harshagurnani/CaDataAnalysis_GoC. Scripts for analyses of preprocessed data and generating figures included in the paper are available at https://github.com/harshagurnani/GoCPopImaging. The latest SilverLab LabVIEW Imaging Software used to acquire the data is available on GitHub at https://github.com/SilverLabUCL/SilverLab-Microscope-Software. NeuroML descriptions of channel and synaptic mechanisms, as well as pre-generated networks and input spike trains are available along with Python scripts for generating network simulations at https://github.com/harshagurnani/GoC_Network_Sim_BehInputs.

### Experimental model and subject details

#### Animals and viral constructs

All animal procedures were carried out in accordance with institutional animal welfare guidelines and the United Kingdom Home Office Animals (Scientific Procedures) Act of 1986. The genetically-encoded calcium indicator GCaMP6f (Chen et al., 2013) was expressed in glycinergic inhibitory interneurons in the granule cell layer. A floxed construct (*AAV9.CAG.flex.GCaMP6f.WPRE.SV40*; Addgene Cat#100835-AAV9) was delivered via microinjection in either lobule IV/V (vermis) or Crus I/II (right hemisphere) in *GlyT2-Cr*e adult transgenic mice. This tranduction strategy labeled 65%–85% of all Golgi cells (GoCs; Figure S1), which are the main interneuron type in the granule cell layer (Simat et al., 2007), along with Lugaro cells and globular cells, which were largely excluded from imaging based on morphological (highly elongated soma or axons extending into the molecular layer) and anatomical (cells close to the Purkinje cell layer) features. All experiments, including surgical procedures and imaging, were performed in adult mice of either sex (6-24 weeks; N = 6 males, 6 females). For immunolabeling experiments, three adult male GlyT2-GFP mice were additionally used.

### Method details

#### Surgical procedures

All surgical procedures were carried out under sterile conditions, with separate procedures for viral delivery and cranial window preparation. Prior to surgery, mice received subcutaneous injections of dexamethasone (1 mg/kg), atropine (0.04 mg/kg) and carpofen (5 mg/kg). Anaesthesia was induced by intraperitoneal injection of a mixture of fentanyl (0.075 mg/kg), medetomidine (0.75 mg/kg) and midazolam (7.5 mg/kg). Reflexes were monitored throughout the surgery, body temperature maintained at 37°C using a regulated heating pad (FHC Inc.), and eyes covered by Viscotears eye gel to prevent dehydration. Post-surgery analgesia (buprenorphine 0.1 mg/kg) was subcutaneously administered prior to anesthesia reversal via atipamezole (3.75 mg/kg), flumazenil (0.75 mg/kg) and naloxone (1.8 40 mg/kg). Animals were kept in a heated chamber until full recovery of reflexes and locomotion, and provided post-operative care and analgesia (buprenorphine 0.1 mg/kg) for 48 hours.

For viral delivery, stereotaxic coordinates were identified based on Allen Mouse Brain Atlas (Crus I/II: (AP) 6.3-6.7 mm from bregma, (ML) 2.4-2.7 mm from midline; (Lob IV/V): (AP) 6.1-6.3 mm from bregma, (ML) ± 0.2 mm from midline) along with visual adjustment based on vasculature and cranial sutures. A small craniotomy was performed at 1-3 sites per animal (to maximize targeting efficiency), and a glass pipette (40-60 μm diameter) preloaded with the viral vector was slowly lowered into the identified coordinates. A pressure-based delivery system (Toohey Spritzer) was used to slowly inject 150-250 nL over 10-15 min, at a depth of 200-400 μm below the pial surface. In five animals this was accompanied by red fluorescent microbeads (4 μm fluospheres, ThermoFisher, 1:2000 dilution) for real time 3D movement correction (Griffiths et al., 2020). 3-6 weeks post AAV expression, a separate surgery was performed to prepare a cranial window for imaging. Part of the neck muscles were dissected, cranial surface was cleared of connective tissue, and a custom-made head plate affixed using cyanoacrylate glue and dental acrylic cement (Paladur, Kulzer), centered over injection coordinates. A 4 mm craniotomy was performed (with occasional ∼1 mm durotomy) and the exposed surface cleared of blood and debris using sterile cortex buffer (125 mM NaCl, 5 mM KCl, 10 mM glucose, 10 mM HEPES, 2 mM MgSO_4_, 2 mM CaCl_2_ [pH 7.4]). The craniotomy was then sealed with a 4 mm diameter glass coverslip (630-2112 VWR) and fixed with cyanoacrylate glue to allow chronic imaging.

#### *In vivo* 3D imaging

Mice were accustomed to head-fixation and the imaging setup (at least 3 sessions, 1 week) before data acquisition. Calcium imaging was performed using a in-house custom acousto-optic lens (AOL) 3D random-access two-photon microscope (Fernández-Alfonso et al., 2014; Kirkby et al., 2010; Nadella et al., 2016) that utilized some components from a Slicescope (Scientifica), a water objective (20 X, 1.0 NA, Olympus) and a 920 nm excitation source (Chameleon Vision, Coherent). The illumination power was typically 10-20 mW, and emitted fluorescence was collected using GaAsP photomultiplier tubes (PMTs, Hamamatsu), in red and green channels (Dichroic: 575dcxr, filters: HQ 525/70 m-2P, HQ 630/100 m-2P). The microscope was controlled with custom SilverLab imaging software (LabView, National Instruments).

A high resolution AOL-based Zstack was first acquired to locate GCaMP6f-expressing neurons within the imaging volume (typically 250 μm x 250 μm x 200 μm, but up to 400 μm x 400 μm x 400 μm). Small imaging ‘patches’ (∼40 μm x 20 μm, pixel size ∼0.5 μm) were manually placed around GoC soma distributed throughout the imaging volume; Video S1). Patches were then selectively imaged with AOL-based random access line scanning. In a subset of experiments (n = 2, N = 2 mice), real time 3D movement correction was performed by tracking a red fluorescent bead (Figure S11; Video S3; Griffiths et al., 2020). Imaging of somatic patches was performed at 100-200 ns voxel dwell times, for a total of 5-25 min over multiple sets of ‘trials’ (10-20 s per trial, 10-30 trials per set) that were subsequently pooled (trials were continuous and only used for optimal memory usage and file storage). Acquisition rate for imaging selected patches varied between 18-97 Hz, depending on the total number. Mice were free to run on a cylindrical treadmill and to whisk. Locomotion was measured using a rotary encoder attached to the wheel (RI58, Hengstler, Germany), and video cameras monitored orofacial movements (300 Hz, Mako) and forelimb/facial areas (30 Hz, The Imaging Source), using a far IR LED for illumination. In n = 3 additional mice, forelimbs were monitored with two faster cameras (300Hz, Mako and 110 Hz, The Imaging Source). A picospritzer was used to deliver brief (100ms), minimally-aversive airpuff to the distal end of the (ipsilateral) whiskers on 1-2 blocks of trials (20 trials/set).

Video S3. Acousto-optic lens 3D two-photon patch imaging of Golgi cell somatic activity in Crus I of an awake, head-fixed mouse with real-time 3D motion correction, related to STAR Methods Video of 25 simultaneously imaged patches (15 μm × 23 μm) with GoC soma expressing GCaMP6f, acquired with realtime 3D movement correction. Locomotion trace shown below with a moving red bar to indicate locomotion aligned with the imaging time points. The acquisition rate was 22 Hz (1×).

#### Immunohistochemistry

GlyT2-GFP (N = 3) and GlyT2-Cre (N = 3, with viral transduction of GCaMP6f) mice were deeply anaesthetised with sodium pentobarbital and transcardially perfused with 4% paraformaldehyde in phosphate buffered saline (PBS). Brains were extracted and post-fixed for 24 h. 70 μm horizontal sections (or as parallel to imaging plane as could be estimated) were prepared using a vibrating microtome (VT1000S, Leica Microsystems) and transferred to multiwell plates for free float immunolabeling. Nonspecific binding was blocked with 5% normal goat serum. Sections were incubated overnight with antibodies for neurogranin (1:500 dilution, AB5620, Millipore), then incubated with a secondary antibody conjugated with Alexa 568 (AB175471, Abcam). Sections were slide mounted with antifade mounting medium and imaged with a Leica TCS SPE8 confocal microscope. Identification of single and double labeled cells was done manually using ImageJ.

#### Extracting somatic fluorescence traces

All analyses were performed using custom scripts in MATLAB or publicly available toolboxes. Before extracting fluorescent traces, post hoc movement correction was performed by maximizing cross correlation with a reference image. Segmentation to obtain spatial footprint of each soma was then performed by a custom procedure, with edge detection on the mean image, followed by detecting maximal closed, connected components. All pixels within the mask were averaged to give raw somatic fluorescence. Normalization was performed per soma using the 10th percentile of raw fluorescence as baseline fluorescence (F_0_) to compute ΔF/F as (F-F_0_)/F_0_. These traces were temporally smoothed using a 100 ms square filter. Further analyses were carried out using this smoothed ΔF/F as GoC somatic activity.

#### Event extraction

Inferred spike ‘events’ were detected from raw fluorescence using a previously published and benchmarked spike estimation algorithm (Berens et al., 2018; Deneux et al., 2016). The algorithm fits a probabilistic model that incorporates slow drift, GCaMP6f kinetics and measurement noise, and returns the most likely spiketrain under model parameters. First, we subselected the cells with high SNR (> 1.8, approximately 85% of recorded cells), followed by visual inspection to remove cells with no isolated transients. SNR for a given cell was defined as the 90th percentile of z-scored responses. Rather than applying fixed model parameters across experiments, we adapted them to each somatic region of interest (ROI). The raw fluorescence was first scaled between 0 and 1, and the slow baseline drift estimate permitted to vary maximally between [0,1]. We used the auto-calibration procedure provided with the toolbox to get a first estimate for the main parameters (decay *tau,* single spike transient amplitude (*a*), and noise magnitude (*sigma*)). To reduce detection of spurious single spikes, the transient amplitude was increased by a factor of 1.1, and noise magnitude increased by a factor of 1.2. We verified the stability of the output by measuring spike count correlation for small deviations of *sigma* and visual inspection of all outputs. For noisier sessions, the auto-calibration gave significant underestimates, and we used a more conservative estimate of *sigma* based on the standard deviation of fluorescence during quiet, resting state of the animal. As single spikes are difficult to resolve from noise with GCaMP6f (Chen et al., 2013), we refer to the resulting spike output as events or event rates (after smoothing in 200 ms boxcar).

#### Behavioral analyses

Running speed was calculated from the angular rotation of the cylindrical wheel, and smoothed with a 100 ms boxcar filter. Motion energy in the whisker pad region (whisker motion index, WMI) was calculated from 300 Hz movies, using the squared frame-to-frame difference in intensity across pixels in regions of interest. For whisking kinematics, we measured the (ipsilateral) whisker angle (Φ), whisking amplitude (W-Amp), and whisker set point (WSP; low frequency changes in whisker angle around which the whisker is positioned or oscillates). DeepLabCut (Mathis et al., 2018; Nath et al., 2019) was used to track 3-4 whiskers (typically row C, 3 points per whisker). As all points share the same underlying model, tracking multiple whiskers (and including the whisker skeleton) generally gave better training and tracking. Whisker angle for each whisker was defined as the angle between a line parallel to the whisker pad, and a linear fit to the 3 points. Angles measured from different whiskers were generally correlated, so we only retained the whisker that had the best tracking performance (estimate likelihood > 0.95 for most time points). This was denoised using a 30 Hz 4th order forward-backward Butterworth filter, and used as the whisker angle (Φ). The whisking amplitude (W-Amp) was measured as the magnitude of Hilbert transform of the filtered angle. The WSP was obtained by Gaussian smoothing of the whisker angle using a 500 ms window. Sessions with poor tracking performance were excluded from the relevant analyses. Ipsilateral paw (wrist and 2 digits) was tracked with front (300Hz) and side (110Hz) view cameras using DeepLabcut, and paw height from the wheel and lateral velocity extracted (Figure S8). The wrist position was used as the most stable indicator of paw position. Times of maximum lift during swing were obtained by identifying prominent peaks in paw height during running epochs. Times of push-off during the end of stance were identified as peaks in lateral velocity during running epochs. Paw lifted toward face or ear regions, or limb movements without sustained wheel movement were distinguished as grooming/non-running epochs.

#### Identification of active periods of behavior and binary state variable

The binary state variable denoted whether the animal was in a behaviorally active period (1), or in a quiet wakeful state (0) for each time point. Active periods were detected using local averages (in 100 ms bins) of whisker motion index (WMI) and locomotion (Loco). Onsets and offsets of individual periods were detected by first upward and downward crossings by either behavioral trace of pre-set thresholds (Onset: 0.25 for WMI, 0.4 for Loco; Offset: 0.2 for WMI, 0.1 for Loco). Detected active periods separated by less than 1 s were merged, and any active period of duration less than 1 s was discarded. For regression analyses in active periods, we included 500 ms before and after these identified periods.

#### Biologically detailed network model

Golgi cell network models were written in NeuroML2 (Cannon et al., 2014; Vella et al., 2014) using Python library *pyneuroml* and simulated using NEURON extension with Python (Hines et al., 2009). A 500 μm x 500 μm x 100 μm volume of the granule cell layer was modeled with a network of 115 GoCs based on measured anatomical density in rodents (Vervaeke et al., 2010). GoCs were modeled as conductance-based multi-compartmental neurons with a reduced morphology, which was previously optimized to have similar behavior as experimentally measured morphologies (Piasini, 2015). Golgi cell ionic conductances were based on published models (Solinas et al., 2007; Vervaeke et al., 2010), and active conductances were restricted to the somatic compartment (Vervaeke et al., 2012). Heterogeneous populations of GoC were created by sampling from 25 sets of channel densities (5 per simulated network), each of which were optimized to have experimentally matched intrinsic firing frequencies (3-9 Hz) and slopes of firing rate-current relationships (14-25 Hz/nA) (Lanore et al., 2019). GoCs were electrically coupled (except where specified) with ohmic gap junctional conductances (0.9 nS for each gap junction; Szoboszlay et al., 2016). These were distributed over the dendritic tree and the probability of electrical coupling between 2 GoCs with intersomatic distance d was given by (Dugué et al., 2009; Vervaeke et al., 2010):

$$P\left(d\right)\;=\;H\left(\;0.01*\;(-1745\;+\;1836/\left(1+exp\left(\frac{d-267}{39}\right)\right)\right)\;$$

where H(x)={xif0≤x≤1;1ifx>1;0otherwise}. For generating different networks, a random number X was sampled uniformly from [0,1] and the 2 cells were connected if X<P(d).

For each connected pair, the total GJ conductance was also a function of intersomatic distance given by:

$$g\left(d\right)\;=g_{0}\;*\;round\left(k\;*\;Y\left(d\right)/5\right),$$

where,

$$Y\left(d\right)\;=\;-2.3\;+\;29.7*exp\left(-\frac{d}{70.4}\right)\;,$$

and $g_{0}=0.9\;nS$, the conductance of a single GJ. This gave a mean total GJ conductance per cell of 22 nS. For testing robustness of population dynamics, the strength of electrical coupling was changed by changing conductance by the scaling factor $k\;\left(≅g/g_{0}\right)$ in the above equations (with k=1 for physiological levels of coupling).

Mossy fibers and granule cells were modeled as spiking input populations, that made synapses onto somatic and apical dendritic compartments, respectively. Synaptic conductances were modeled as AMPAR type conductances based on measured EPSC kinetics (Cesana et al., 2013; Kanichay and Silver, 2008), with weighted double and single exponential decays, respectively.

**Table:** 

Synapse	Reversal potential (mV)	τ_rise_ (ms)	g_1_ (nS)	τ_d1_ (ms)	g_2_ (nS)	τ_d2_ (ms)	Peak conductance (nS)
MF-GoC	0	0.1	0.7	0.7	0.2	3.5	0.89
PF-GoC	0	0.1	0.67	1.06	-	-	0.67

#### Spiking input populations

Input spikes were drawn from an inhomogeneous Poisson process, with time-varying mean firing rates $\left\{r_{k}\left(t\right)\right\}$ generated as a weighted linear combination of simultaneous “behavioural measures” ($\bar{b}\left(t\right)$), with additional scaling (A) and offset (c):

$$r_{k}\left(t\right)\;=A\;{\bar{w}}_{k}\;*\;\bar{b}\left(t\right)\;+\;c$$

where $\bar{b}\left(t\right)$ is 6-dimensional behavioral activity from an experimental session, defined by the following variables (normalized): binary state, locomotion, WSP, WMI, WAmp, pupil area. A fixed 20 s segment of $\bar{b}\left(t\right)$ was used, except where otherwise specified, with different randomly-drawn sparse weight vectors ${\bar{w}}_{k}$ to generate a population of input *spike arrays*. To add inputs uncorrelated to “ongoing behaviour,” a different random 20 s segment of the behavioral recordings was selected for each additional input. In addition, a low-firing background input population (30 MFs at 5Hz, 60 PFs at 2Hz; spikes generated as a Poisson process) was included for all simulations.

#### Properties of modeled excitatory synaptic inputs

Four different kinds of input connectivity were examined, with 16-27 different input levels for each of the four conditions, and 3 different GoC populations per input level:

(1) Behaviorally-driven inputs with random connectivity (“Input-Beh”):
For each input k, its firing rate $r_{k}\left(t\right)$ was determined by ${\bar{w}}_{k}=\left(w_{1k},\;\ldots ,w_{6k}\right)$, with each $w_{jk}$ drawn uniformly from [0,1] and set to 0 with probability 0.5 (to make $\bar{w}$ sparse). Scaling (A) and offset (c) was set to 50 Hz and 7 Hz respectively, and firing rates clamped below at 2 Hz. Number of MFs and PFs were independently varied between 0-48 and 0-120 respectively, each of which were randomly distributed in the volume and could synapse with fixed probability of 0.2 on any GoC within a specified neighborhood (within 300 μm for MFs, upto 100 μm along x axis for PFs).
(2) Two populations of behaviorally-driven inputs with random connectivity (“Bimodal inputs”):
Two different populations were randomly distributed in the volume, with the first population drawn from the same distribution as *Input-Beh.* For the second population, scaling was set to - 25 Hz, and offset set to 30 Hz. This modeled populations of positively and negatively modulated inputs based on experimental observations (Roš et al., Cosyne abstract 2018; Lanore et al., 2021). Fraction and numbers of these input populations were varied for different simulations. Connectivity was random, as described for *Input-Beh.*
(3) Additional uncorrelated inputs, random connectivity (“Uncorrelated inputs”):
Additional inputs were generated with identical weight distribution as *Input-Beh*, but using different segments of behavioral activity (different for each input, same segment of each behavioral variable for a given input). The number of these additional uncorrelated inputs varied between 20%–200% of the number of correlated inputs (*Input-Beh*) and changed the eigenspectrum of the inputs. Connectivity was random, as described for *Input-Beh.*
(4) Clustered connectivity with pure behavioral inputs (“Tuned inputs”):
The volume was divided into four sagittal “modules,” of 125 μm width each. Each module received inputs with firing rates mainly determined by a single behavioral variable (Loco, WMI+WAmp, Pupil area, WSP), and connectivity further restricted to within 125 μm for MFs. Input populations were created by choosing weight vectors with one dominant weight sampled uniformly from [0.5,1] and other weights sampled from [-0.1,0.1], with each weight then set to 0 with probability 0.3. The dominant behavioral identity was randomly permuted across the 4 modules for different simulations. The inputs showed higher within-module correlations and reduced across-module correlations, and thereby decreased the spatial scale of input correlations between GoCs.

Each input connectivity scheme was tested with experimentally-constrained gap junction coupling between GoCs (“GJs”) or without electrical coupling (*“no GJs”*).

#### Measuring somatic activity and analysis of model behavior

All simulations were performed with 0.025 ms numerical integration timestep. To avoid storing voltage traces at such high “acquisition” rates beyond the purposes of model validation, only the spike times of each GoC and input were saved. Spikes were converted to firing rates by binning in 40 ms time bins, which was further used for all subsequent analyses. For comparing measures of population activity to those of experimental data, GoC sub-populations were subsampled from smaller volumes (300 μm x 300 μm x 100 μm; 35-40 cells), and measures averaged across 10 such sub-populations for each simulation.

### Quantification and statistical analysis

#### Correlations

Pairwise correlations between GoCs were calculated in 40 ms bins on ΔF/F traces (total correlation) or after projecting out the first population mode (residual correlations). To check significance, correlation was recalculated after shuffling traces by circularly permuting activity (independently for different neurons). This was repeated 500 times to calculate a null distribution of correlations for each pair. The pair was considered to have significant positive correlation if it exceeded 97.5th percentile of the null distribution for that pair, and to have negative correlation if it was lower than the 2.5th percentile. Similar analyses were performed for event rates (events smoothed in 200 ms bins).

To measure dependence on pairwise distance between GoCs (d), correlations were averaged over 20 μm bins, and fit with a linear function (total correlations), or exponential function (residual correlations):

$$\rho \left(d\right)\;=\;A\cdot d\;+b\;\text{Linear}\;\text{fit}\;\text{for}\;\text{total}\;\text{correlation}$$

$$\rho \left(d\right)=\;A\;exp\left(-\frac{d}{\lambda }\right)+b\;\text{Exponential}\;\text{fit}\;\text{for}\;\text{residual}\;\text{correlation}$$

This was done separately for all residual correlations, and pairs with significant positive and negative residual correlation. The first 20 μm were excluded from analyses to avoid contamination from fluorescence arising from the soma of an individual cell.

#### Calculation of population modes and residual activity

Singular value decomposition (SVD) was used to decompose (zero-centered) neural population activity into orthogonal population modes: $X\;=\;USV^{T}$, where S is a diagonal matrix of covariance along different modes (eigenvalues of covariance matrix, visualized in Figure 2B), U is the loadings matrix, V is the dynamics along different modes and where $V^{T}$ is the transpose of V.

Residual activity of each neuron was calculated by projecting out the top mode (i.e., projecting the activity on the N-1 dimensional space excluding PM1). This was done by first decomposing the population activity using SVD, and then retaining all but the first mode to reconstitute residual activity:

$$\;X_{res}\;=\;U_{∼1}\cdot S_{∼1}\cdot V_{∼1}^{T}$$

where $U_{∼1}$and $V_{∼1}$ only have columns 2 to N of U and V respectively, and $S_{∼1}$ only has the corresponding N-1 rows and columns.

#### Identification of shared, reliable modes

For assessing the contribution of different modes and identifying *reliable* population modes (i.e., shared), we used bi-cross-validation after decomposing activity into modes with SVD (Owen and Perry, 2009; Stringer et al., 2019). The data was divided into training (70%) and test (30%) sets, and the training data decomposed to find the loadings (U). We then retained the top K modes, and the corresponding loadings ($U^{K}$, first K columns of U) to quantify the explained variance by this K-dimensional reconstruction of the population activity. We further split the test data into a second partition of training neurons ($X_{1}$, 80 % of the population) and test neurons ($X_{2}$, 20% of the population). The low-rank approximation for the entire test data can be written in block format:

$$\left[X_{1}\;;\;X_{2}\right]\;\approx \;\left[U_{1}^{K};\;U_{2}^{K}\;\right]\;S_{te}V_{te}^{T}$$

We use the upper block to estimate the K latent dynamics $\left(S_{te}V_{te}^{T}\right)$ via linear regression, and use the lower block to get a rank-K estimate for $X_{2}$:

$$\hat{X_{2}}\;=\;U_{2}^{K}\cdot \left({\hat{S}}_{te}\;{\hat{V}}_{te}^{T}\right)\;=\;U_{2}^{K}\;\cdot pinv\left(U_{1}^{K}\right)\;\cdot X_{1}$$

where pinv is the Moore-Penrose pseudoinverse. The average explained variance by the reconstruction of test data was computed for 30 repeats with different training/test partitions.

#### Dimensionality

To estimate the number of reliable population modes in the population activity we used the cross-validated explained variance (CVEV) on test data, as defined above. CVEV increased with stable population modes, and started decreasing once it was overfit to training data. Thus the peak of the relationship between explained variance and number of modes (*K*) provides a lower limit for the number of reliable population modes (*shared* dimensionality) and the maximal explainable variance for a given recording. This method weighted large and weak modes equally (similar to a threshold method), as long as there was significant generalization to test data. Due to the requirement of generalization to held-out neurons, this only captures modes that are shared between at least a few GoCs (*i.e., shared subspace*), and cannot distinguish independent measurement noise from reliable but private variability of individual GoCs. It is thus a lower bound for the dimensionality of the total GoC coding space, limited by SNR and shared variability.

We also used a different definition of dimensionality that reflects the entire eigenvalue distribution along different modes (Abbott et al., 2011). The effective dimensionality $\left(D_{eff}\right)$, also sometimes referred to as ‘participation ratio’, was defined as

$$D_{eff}\;=\;{\left(\overset{K}{\underset{i=1}{\sum }}{\hat{\lambda }}_{i}^{2}\right)}^{-1}\;\text{ where}\;{\hat{\lambda }}_{i}^{2}=\frac{\lambda_{i}^{2}}{{\left(\sum_{i=1}^{K}\lambda_{i}\right)}^{2}}\;$$

and λ_i_ is an eigenvalue of the covariance matrix. If the variance is equally distributed along ***M*** modes (and all other modes have zero variance), then $D_{eff}=$ ***M***. If, instead, one mode captures most of the variance, $D_{eff}$ will be close to 1. This includes both shared and independent variability but is sensitive to the relative amplitudes of different modes. It cannot exclude measurement noise, but may be more suitable for a stricter lower bound on net input variability in the case of homogeneous downstream connectivity and ‘linear’ postsynaptic responses.

#### Similarity of loadings

To compute similarity of neural activity accounted for by ‘signal’ modes (i.e., all population modes upto the peak of CVEV curves), we computed the dot product of the corresponding loadings (excluding the large PM1) onto each pair of neurons (Figures 6E and S7E). Specifically, for a recording with signal dimensionality of K, we took the columns 2 to K of the loadings (U), where each row $\left(u_{m}^{2:K}\right)$ corresponds to selected loadings for a single neuron. The response similarity (overlap) between 2 neurons was given by $u_{1}^{2:K}\cdot \;u_{2}^{2:K}$.

#### Population modes using independent-component analysis

To examine the structure of population modes identified without the constraint of Gaussian variability or orthogonal modes, we used independent component analysis (ICA) to decompose activity into a specified number of components (which allows for activity along modes to be heavy-tailed). To estimate the minimum dimensionality of the neural population activity, we computed the stability of identified mode loadings on different partitions of the dataset. On each iteration, ICA was performed on two halves of a random partition of the data (time points divided in 500 ms blocks). The dot product between the loadings from the two decompositions was used to align each mode with its maximally correlated mode from the other half. The average correlation between these aligned loadings was computed for each iteration, and averaged across 10 such partitions for each specified number of modes. The minimal dimensionality was chosen such that successive average stability values i.e., average correlation if data was decomposed into more modes, were all less than 0.9. This conservative estimate was used to determine the analyses of variance of loadings for reliable ‘signal’ modes, and compute angles within these stable subspaces. For angles between identified modes, the first principle angle was calculated between selected mode (PM1 or non-PM1) and the subspace defined by the remaining ‘signal’ modes.

#### Linear regression of population modes and behavior

Cross-validated linear regression was used to assess how well different behaviors explained the dynamics of the global population mode (PM1), as well as whether heterogeneous GoC activity contained further behavioral information. All state and kinematic variables, and dynamics of the population modes were restricted to behaviorally active periods ± 500 ms, and downsampled to the slowest acquisition rate of 20 Hz for the analysis. Data was partitioned into training (80% of time points, randomly sampled in 500 ms blocks) and test data (20% of time points), and regression coefficients determined using L2 regularisation with mean squared error. This was repeated for 100 different partitions of the data to give mean CVEV for each session/behavior. To examine what explained the variability along PM1 (Figure 3), regression of PM1 was performed against individual behavioral variables, or all behaviors simultaneously (multilinear regression, *LinearComb*). Model comparison was possible despite the different number of parameters, as we used cross-validated performance, which was optimized by using L2 regularisation to assess model performance and guard against overfitting of the data.

A similar cross-validation procedure was used to decode individual behavioral variables from different low rank decompositions of the population activity (Figure 7A). For a given partition of data, regression was repeated for rank 1 to rank N approximation of population activity, where rank K is given by retaining only the top K modes (initial SVD performed on all time points). The performance was compared for rank 1 (only common mode, PM1) to the optimal number of modes (maximum average cross-validated explained variance). To further confirm that the observed increase in behavioral information was specific to ongoing behavioral dynamics, we performed two controls by repeating regression for shuffled behavioral variables (in 500 ms blocks during the active periods only) and measuring the maximal explained variance. For within-period shuffle, the 500 ms blocks from the same active period were rearranged, which maintains average behavioral activity or general context, and optimal performance was expected to be comparable to PM1 on real data. For across-period shuffle, the 500 ms blocks were randomized across all active periods (this surrogate only preserves the local behavioral dynamics). The optimal number of differential modes used for decoding were determined as the number of additional modes that increased the CVEV by at least 90% of the maximal increase (as the CVEV curves saturate).

#### Response to air puff

To examine the reliability of GoCs, responses to airpuff were quantified. To determine the timing of responses from GCaMP6f kinetics, we extracted events rather than using the slower ΔF/F, and used responses in 50 ms bins (based on the slowest acquisition rate). Response latency was calculated as the latency of the first event after the onset of airpuff, as well as time of the peak event rate (within 0-800 ms post airpuff). Additionally, the net activity was calculated in the following windows:

Activity before puff (***A1***): −400 ms to −200 ms from puff onset
Activity after puff (***A2***): 0 to 800 ms from puff onset
Early response (***R1***): 0 to 300 ms from puff onset
Late response (***R2***): 300 to 800 ms from puff onset

To calculate the response variability of single neurons (inter-trial variability), we calculated the standard deviation of onset latencies for each neuron, as well as response rates for both early (R1) and late responses (R2) across trials. For the same session, the variability across simultaneously recorded GoCs (intra-trial variability) was computed as standard deviation of responses and latencies across neurons on each trial. For comparision, both inter-trial and intra-trial variability were averaged across all neurons and trials respectively for each session. These session averages were used for paired statistical testing (Wilcoxson signed-rank). To further examine intra-trial variability, we computed pairwise correlations for each session restricted to the airpuff response epochs, by concatenating the puff-evoked activity (0-800 ms) across all trials.

Trial-averaged response to air puff was quantified for each neuron after aligning extracted events to airpuff onset, and were further classified as follows (for GoCs across all sessions):

(1) ***Class I***: Fast, transient response: A2 > A1 and R1-R2 > 0.05 Hz (0.05 Hz determined as chance level)
(2) ***Class II***: Variable delay response: A2 > A1 and R1∼R2 (R1-R2 < 0.05 Hz)
(3) ***Class III***: No/decreased response: A1∼A2 or A1 < A2

Mean latency and inter-trial variability of individual neurons were grouped by class to further report class-specific averages.

To compute reliability of GoC population responses across trials, the event rates of all GoCs within a session were collected into a population response vector $\left({\underline{r}}_{j}\left(t\right)\;=\;\left[r_{1j}\left(t\right),\;r_{2j}\left(t\right)\;,\;\ldots \;,\;r_{nj}\left(t\right)\right]\;\right)$ for each 100 ms time bin (t) aligned to puff onset, for every trial j. The similarity for each time bin was given as the dot product between the corresponding population response vectors for a pair of trials, averaged across all trial pairs.

$$S\left(t\right)\;=\;<{\underline{r}}_{j}\left(t\right)\;\cdot {\underline{r}}_{k}\left(t\right)\;{>}_{j,k}$$

For quantifying similarity of behavioral response across trials, we used whisker motion index, and computed its coefficient of variation across trials, for each puff-aligned time bin. To compare the mean weighted decay timescale of these similarity profiles, we normalized the profiles between 0 to 1, and computed the integral in the 0-800ms window.

#### Modulation of individual Golgi cell activity within step cycle

To examine modulation of GoC activity (inferred events) with ipsilateral forelimb step cycle, we used each instance of paw lift within running epochs, and quantified aligned GoC responses in a ± 1 s window around each lift. The average across all such sessions defined a lift-aligned event probability for each GoC, as well as a mean trajectory of the paw. A control was defined for each GoC, by shuffling events within each instance and then averaging those to give a ‘shuffled event probability’. Pearson correlation was computed between the mean paw trajectory and the event probability $\left(r_{event}\right)$, as well as between the paw trajectory and the shuffled event probability $\left(r_{shuff}\right)$. A GoC was considered significantly modulated within the step cycle if its event probability was highly correlated with the lift $\left(\left|r_{event}\right|>\;0.5,\;p<0.01\right)$ but the shuffle control was not (p>0.1). Similar analyses were performed after aligning to push-phase, using correlation with the lateral velocity.

#### Statistical presentation of data and tests

Unless otherwise specified, data is reported as mean ± SD in text, error bars/area in figures indicate mean ± SEM, and sample sizes are reported as number of sessions (*n*) and number of animals (*N*). Standard least squared-error minimization was used to fit linear and exponential functions. Multiple linear regression for population modes and behavior was performed with L2 regularisation, optimized with CVEV.

Statistical significance was assessed using nonparametric tests - Mann-Whitney U Test for unpaired data and Wilcoxson signed-rank test for paired data. For time-shuffled controls, data was shuffled in 500 ms blocks, and the 95^th^ percentile (2.5^th^ to 97.5^th^) of this distribution was used as the significance level. To test if positively and negatively-correlated populations were differentially distributed, Kolmogorov-Smirnov test was used on equally subsampled data. All sessions were considered independent samples, unless specified.

## Supplemental information

Document S1. Figures S1–S11

Document S2. Article plus supplemental information
