# Supplementary material for: Multidimensional population activity in an electrically coupled inhibitory circuit in the cerebellar cortex
Source: Neuron. 2021 May 19;109(10):1739–1753.e8. doi: 10.1016/j.neuron.2021.03.027 (PMC8153252; doi:10.1016/j.neuron.2021.03.027)
Supplement: Document S1. Figures S1–S11 [file mmc1.pdf]

Neuron, Volume *109*

Supplemental information

Multidimensional population activity in an electrically coupled inhibitory circuit in the cerebellar cortex

Harsha Gurnani and R. Angus Silver

Supplementary Figures

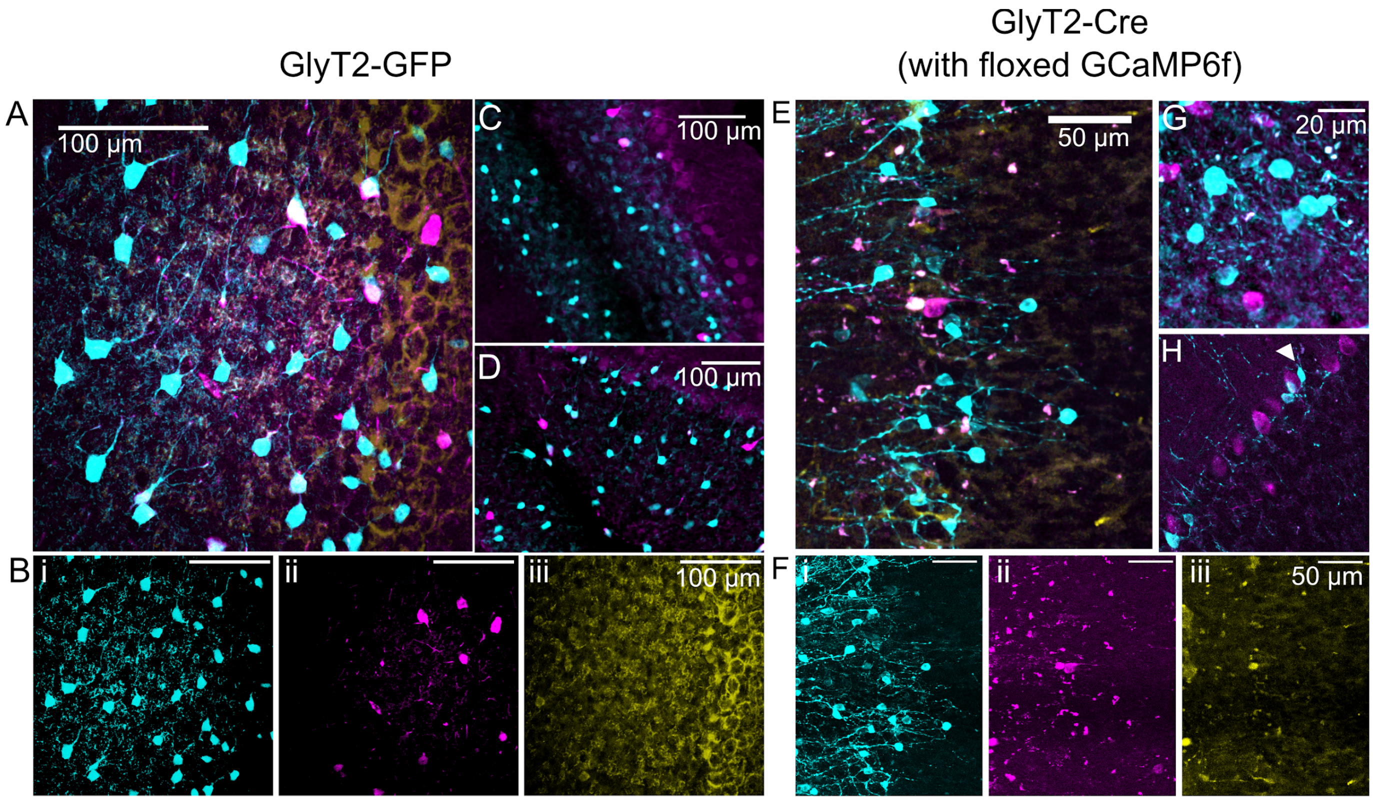

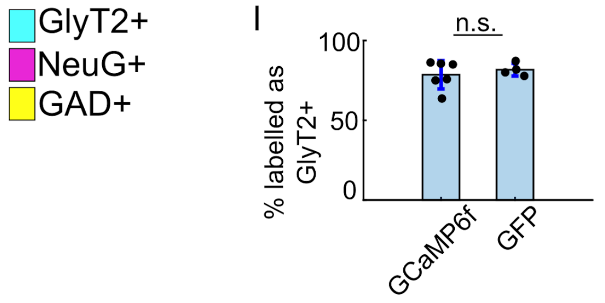

J

Class of GrC layer inhibitory interneuron	Fraction of all identified neurons (GlyT2+ or NeuG+)	Fraction of all GoCs	Fraction of imaged GCaMP6f-expressing cells
GlyT2-positive interneurons	78% (65-87%)	85%	100%
Lugaro cells	10% (5-15%)	-	0 (Rare)
Golgi cells	67%	-	80-92%
Globular cells	17%	-	8-20%

Figure S1: GFP and GCaMP6f expression in glycinergic neurons in the cerebellar input layer - comparison of expression levels with transgenic and viral techniques. Related to Figure 1.

(A-D) Example confocal images (max projection of 70 µm thick section) of cerebellar cortex from GlyT2-GFP transgenic mice, with glycinergic neurons labelled with GFP (cyan), and *post hoc* immunostaining for neurogranin (magenta). Bi-iii - Individual channels for example overlay in A, with additional immunostaining for GAD (yellow). A,B,D - Crus II, C - Lobule IV/V.
(E-H) Example confocal images of cerebellar cortex from GlyT2-Cre animals virally transduced with floxed GCaMP6f (cyan), and *post hoc* immunostaining for neurogranin (NeuG). Fi-iii - Individual channels for example overlay in E, which includes additional immunostaining for GAD (weak labelling of soma). E,F,G - Crus I/II, H - Lobule IV/V with a white arrowhead showing a putative Lugaro cell expressing GCaMP6f (5-15% of all cells). (I) Bar graph showing percentage of GlyT2 positive cells in GlyT2-GFP transgenic (mean=81.5±2.2, n=4/N=3) and GlyT2-Cre (mean=78.3±4, n=6/N=3) mice transduced with flexed GCaMP6f, as a fraction of all inhibitory interneurons. (J) Expected fraction of different granule cell

layer (GCL) inhibitory interneurons, given similar levels of expression in GlyT2-Cre/GCaMP6f and GlyT2-GFP mice in Simat et al, 2007. For *in vivo* imaging sessions, elongated/small morphology, location near the Purkinje cell layer and axon location helped identify and exclude putative Lugaro and globular cells. Values in blue are estimates from current study, values in red were reported in Simat et al, 2007, used for estimates in the last column.

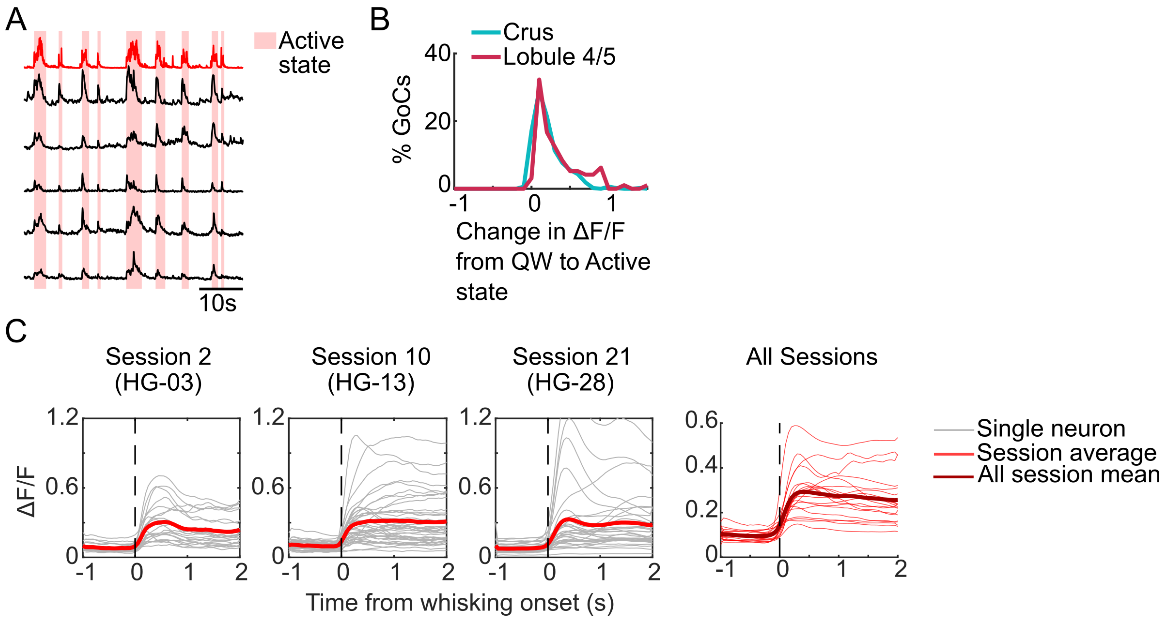

Figure S2: Widespread increase in Golgi cell activity during active state and whisking. Related to Figure 1

(A) Example activity (ΔF/F) of 5 Golgi cells (GoCs, black) aligned with whisker motion index (red trace), with behaviourally active states shaded in light red.

(B) Distribution of change in mean activity from quiet wakeful (QW) to behavioural active state for all GoCs (Crus: n=476 GoCs, N=5 animals, Lob IV/V: n=106/N=4)

(C) Whisking-onset (dashed line) aligned activity for all GoCs (gray) and population average (red) for 3 example sessions, and population average for all sessions (right, n=21 sessions, N=9 animals).

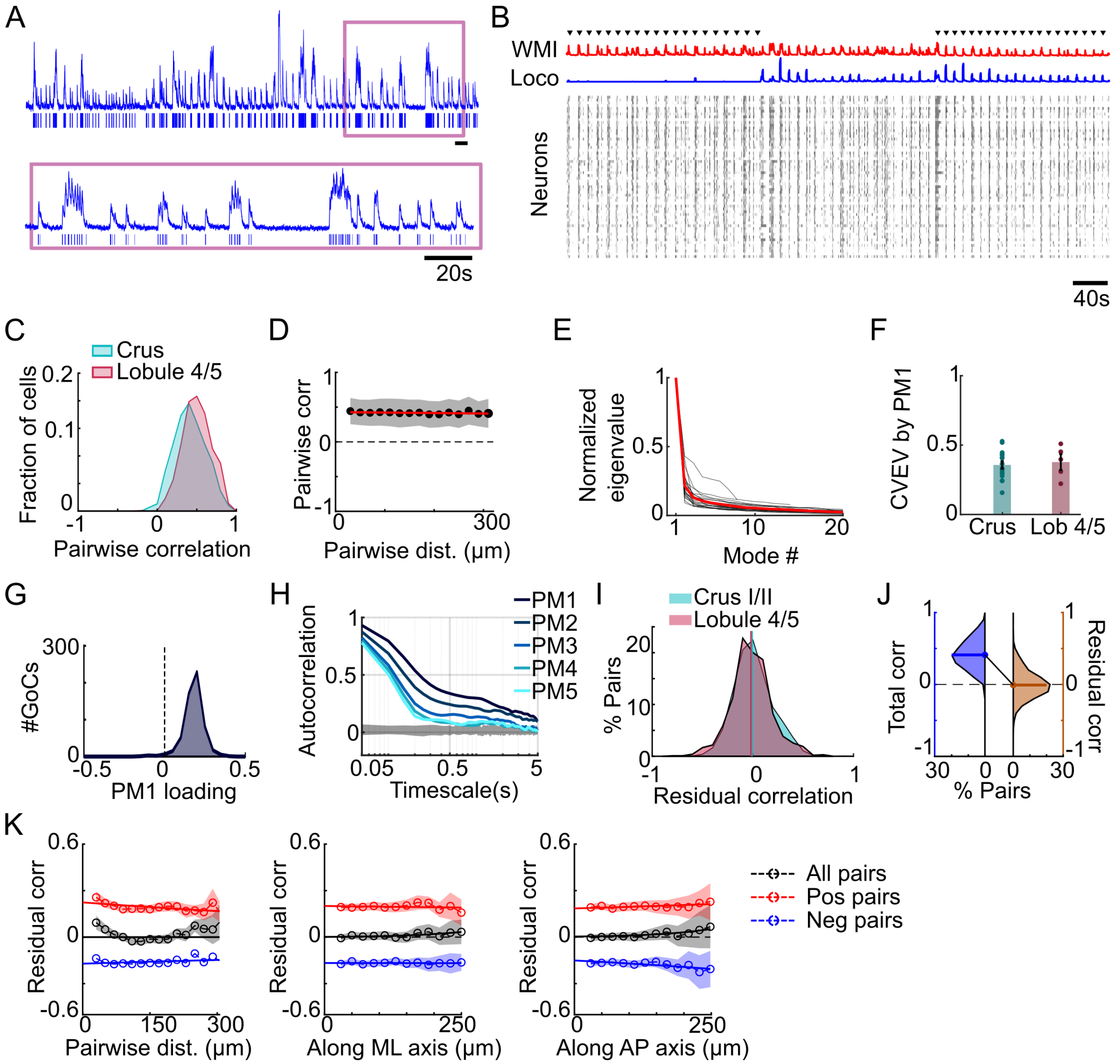

Figure S3: Analyses with inferred spike-related events, Related to Figure 1,2 & 4.

(A) Top: Example ΔF/F (normalised) and inferred underlying spikes (events, vertical lines below) for a single Golgi cell (GoC). Bottom: Expanded timescale for period highlighted by magenta box. Scale bar, 20s.

(B) Extracted events for all GoCs, with whisker motion index (WMI, red), locomotion (Loco, blue) and timing of mild air puff to the whiskers (black triangles).

(C) Distribution of pairwise correlations between GoC event rates for both cerebellar lobules (Crus I/II: n=16 sessions/N=5 animals; Lob IV/V: n=5/N=4)

(D) Distance-dependence of total event correlations. Black line indicates mean across all pairs, Shaded area indicates standard deviation, Red line indicates linear fit.

(E) Eigenspectrum for the population covariance matrix for event rates, normalized by the maximum eigenvalue in each session and arranged by decreasing order of eigenvalue. Black lines are individual sessions (n=21, N=9 mice), solid red line is the mean across sessions.

(F) Cross-validated explained variance (CVEV) by top population mode (PM1) calculated from events for both cerebellar lobules (Crus: n=16/N=5, Lob IV/V: n=5/N=4).

(G) Distribution of loadings of top mode (PM1) for all neurons (n=21/N=9).

(H) Autocorrelation of top 5 population modes for GoC event rates. Darkest to the lightest blue line indicates PM1 to PM5. Gray shaded region indicates confidence interval from the shuffle control.
(I) Distribution of residual correlations in Crus I/II (cyan; n=16/N=5) and Lobule IV/V (magenta; n=5/N=4) after projecting out PM1
(J) Distribution of pairwise correlations for total event rates (blue), and residual activity after projecting out the top population mode (PM1, orange, n=21/N=9).
(K) Dependence of residual correlations of inferred events on net pairwise distance (left), as well as distance along medio-lateral (ML, along parallel fibres; middle) or antero-posterior (AP, orthogonal to parallel fibres; right) axis. Scatter shows average residual correlation (in 20 μm bins), shaded regions indicate mean±SEM, and solid lines denote exponential fits to data.

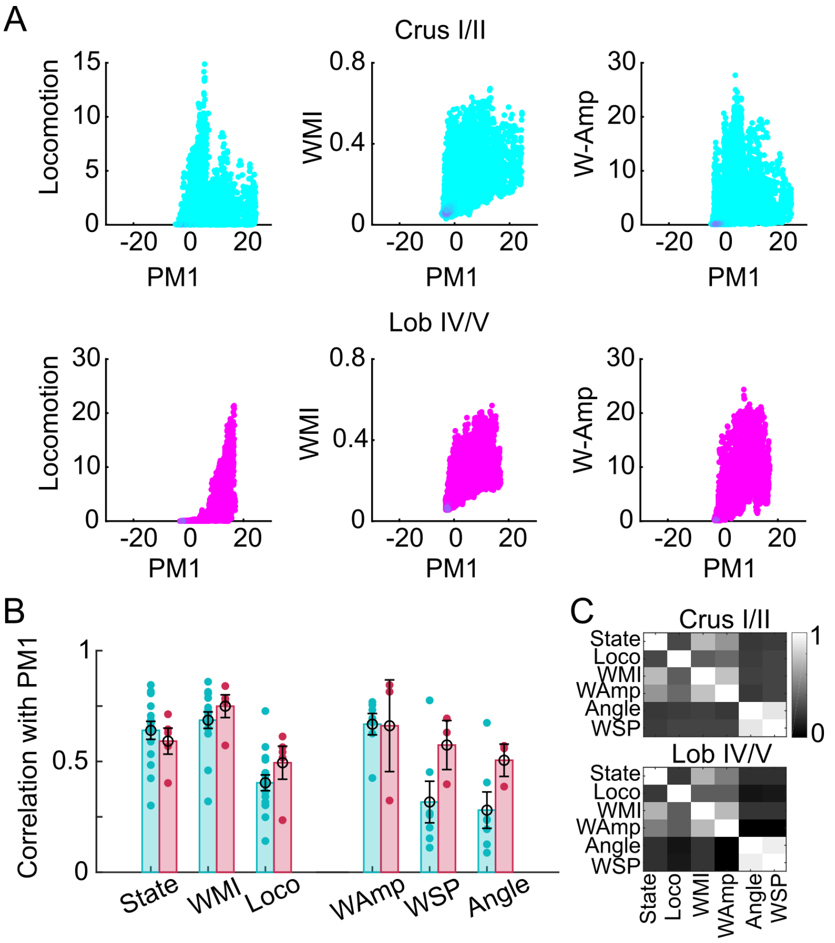

Figure S4: Correlation of population mode 1 with behaviours. Related to Figure 3.

(A) Relationship between behavioural variables (Locomotion, whisker motion index - WMI, whisking amplitude - W-Amp) with population mode 1 (PM1; from ΔF/F) across all timepoints for one session, in Crus I/II (top) and Lob IV/V (bottom) .

(B) Correlation of PM1 with individual behavioural and state variables. Scatter shows individual sessions (Crus: cyan, Lob IV/V: magenta). Bar and error bar indicate mean±SEM across all sessions.

(C) Correlation between different behavioural variables, averaged across all sessions, separately for both lobules. (For state to WMI: n=20, N=9 animals; For WAmp to WSP: n=10/N=7)

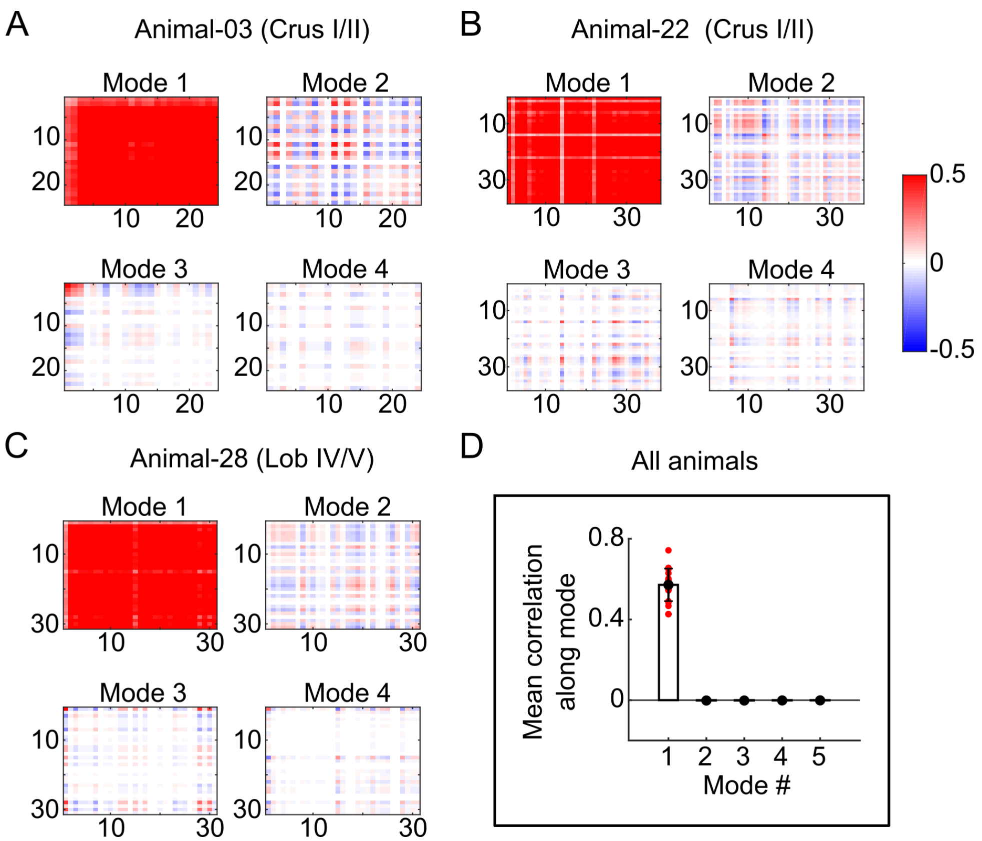

Figure S5: Covariability along different population modes. Related to Figure 4.

(A-C) Covariance matrix along top 4 population modes for example sessions for 3 mice
(D) Mean correlations for the top 4 modes for all sessions and mice. Red scatter denotes individual sessions, and bar with error bar denotes mean±SEM across sessions.

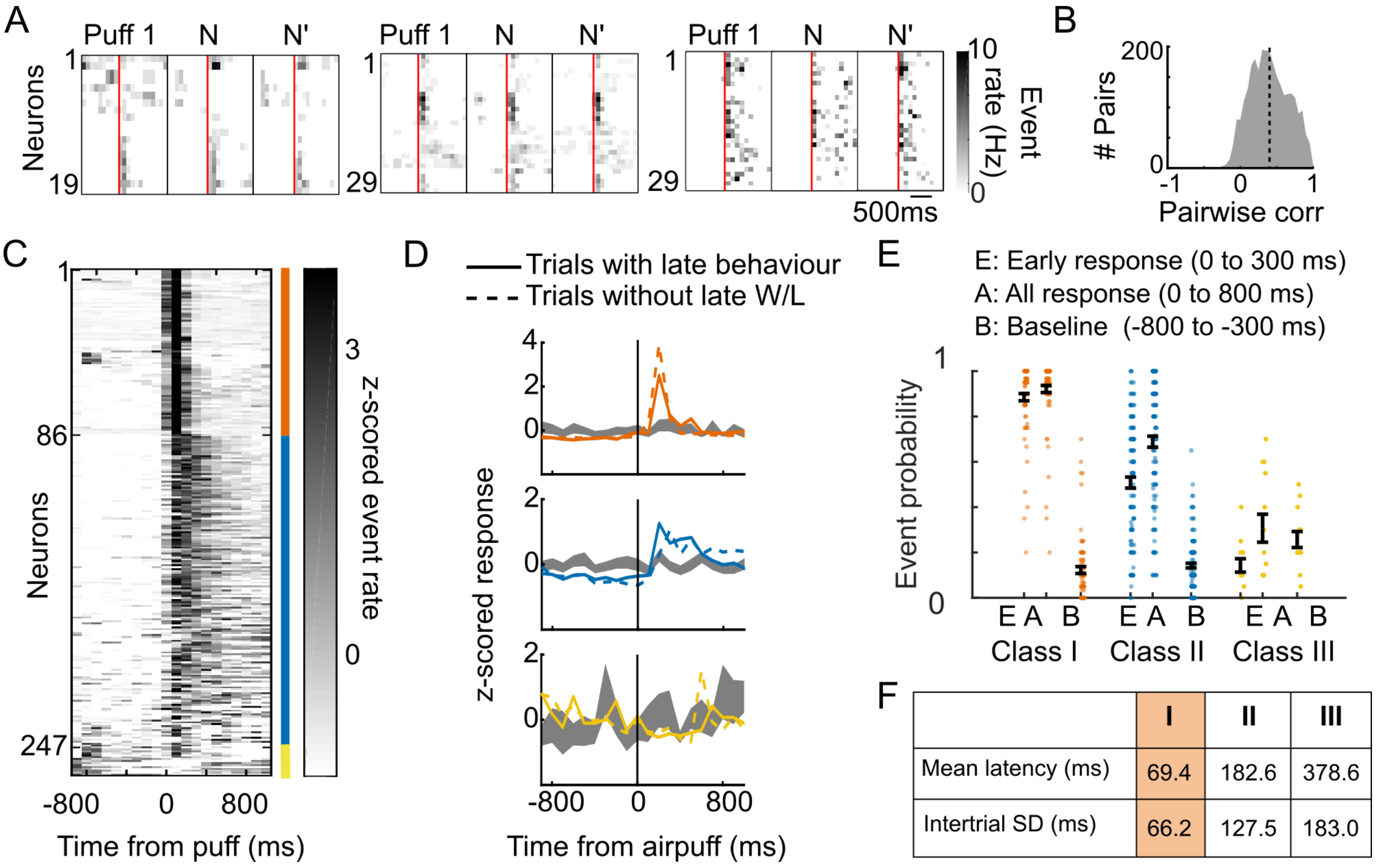

	I	II	III
Mean latency (ms)	69.4	182.6	378.6
Intertrial SD (ms)	66.2	127.5	183.0

Figure S6: Classification and reliability of Golgi cell responses to airpuff to the whiskers. Related to Figure 5.

(A) Single trial responses of Golgi cell (GoC) populations to airpuff (AP) on distal end of ipsilateral whiskers, during 3 random trials, for 3 different populations (sessions). Red line indicates the onset of airpuff.

(B) Distribution of pairwise correlations calculated during all concatenated AP-triggered response epochs within each session (n=12 sessions/N=5 animals).

(C) Trial-averaged responses for all GoCs (n=261 GoCs/N=5 animals). Mean responses were z-scored before plotting on the same scale. GoCs were sorted by response type, as indicated by color of the bar on the side (Class I: orange, Class II: blue, Class III: yellow).

(D) Mean AP-triggered response for the 3 classified types compared to time-shuffled responses (gray), averaged over trials with whisking and locomotion in the late epoch (bold), and over trials without extended behavioural response (dashed).

(E) Summary of individual GoC event probability during early (*E*; 0-300ms) and all AP-triggered response (*A*; 0-800ms) epoch, as well as baseline before AP (*B*; -800 to -300 ms). Mean±SEM for all classes of responses. Class I (n=85), E: 0.89±0.15, A: 0.92±0.14, B:0.13 ±0.14. Class II (n=161), E: 0.51 ±0.28, A: 0.69 ±0.28, B: 0.14 ±0.12. Class III (n=15), E: 0.14 ±0.11, A: 0.30 ±0.22, B: 0.26 ±0.13.

(F) Summary of individual GoC mean latency to first event and its trial-to-trial variability (SD of latency).

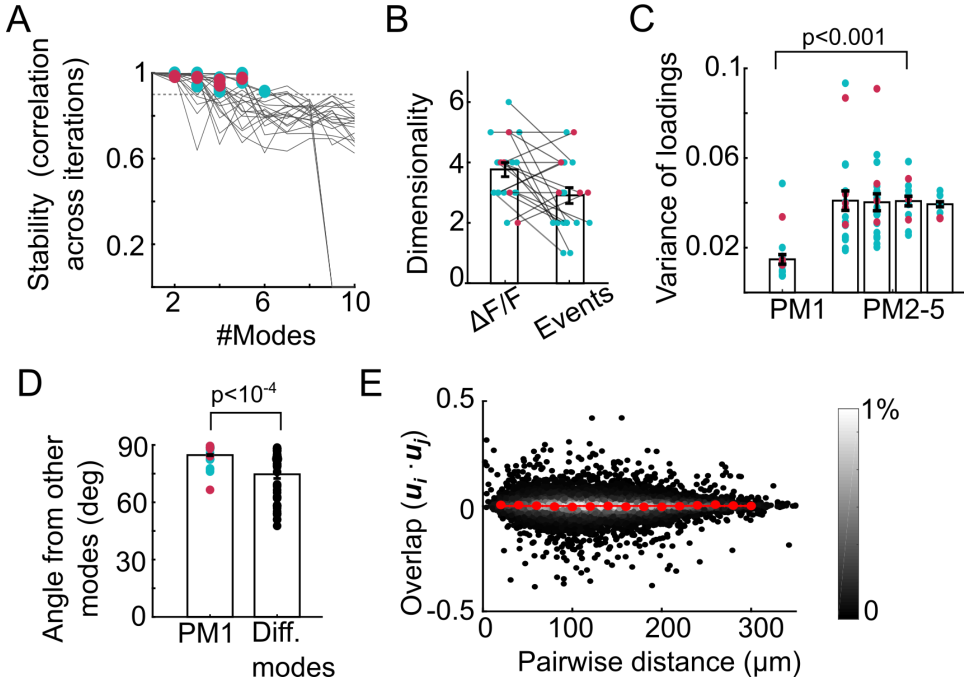

Figure S7: Population modes identified with Independent Component Analysis. Related to Figure 6.

(A) Relationship between average correlation of maximally-aligned loadings identified using ICA on different partitions of the neural recordings (stability) versus number of identified population modes (PM). Grey lines indicate individual sessions and dashed line indicates criterion for 'minimum dimensionality'. Crus I/II (cyan): n=16 sessions,N=5 animals, Lob IV/V (magenta): n=5/N=4.
(B) Minimum dimensionality estimated using ICA for both ΔF/F and event rates. Scatter denotes individual sessions, bar and error bars indicate mean ± SEM across sessions. Same sessions as in A.
(C) Variance across each population for loading corresponding to common (PM1) and differential modes (PM >1). Only loadings for highly reliable PMs (identified in A) were used for comparison. Significant difference between variance of PM1 and other modes ($p<0.001$ for PM1 versus non-PM1, Wilcoxson signed-rank test) suggests that there is a clearly identified common mode.
(D) Angle between individual mode and subspace defined by remaining modes (at the estimated dimensionality). The identified common mode is nearly orthogonal (mean±SD: 84±6°) to differential activity, and significantly larger than angle between differential modes (mean±SD: 74±12°, $p=3 \times 10^{-5}$, Mann Whitney U test).
(E) Similarity between GoCs as measured by overlap (dot product) of their loadings (excluding PM1) as a function of their pairwise distance. Red symbols indicate binned averages and line denotes linear fit. Colorbar indicates probability density.

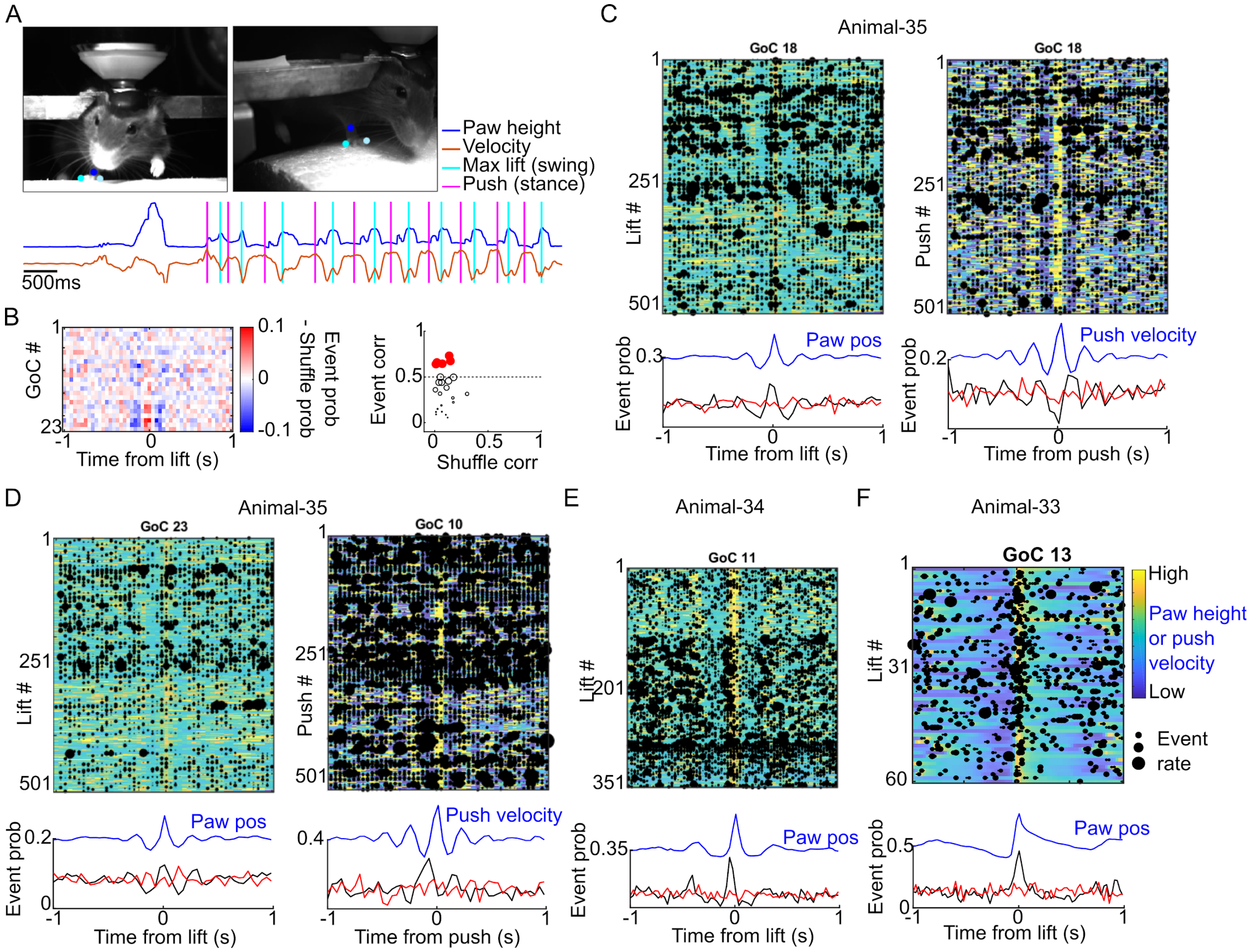

Figure S8: Modulation of the activity of individual Golgi cells during forelimb step cycle. Related to Figure 7.

(A) Top: Example frames from front (300Hz) and side (110Hz) camera with tracking of ipsilateral paw (blue dots) using DeepLabCut. Bottom: Lateral and vertical motion of ipsilateral wrist were used to quantify limb kinematics. Paw height (blue) and push velocity (orange). Times of maximum lift (swing phase) and push (end of stance phase) are indicated by cyan and magenta bars respectively.
(B) Left: Difference in lift-aligned event probability and shuffle control for all GoCs in a session. Right: Correlation of lift-aligned event probability and mean paw height, versus correlation between shuffled probability and paw height (black circles). Size of circle is inversely related to p-value of correlation coefficient. Significantly modulated cells are indicated by filled red circles (true correlation>0.5, p-value for true correlation<0.01, p-value for shuffled event correlation>0.1).
(C) Example of single cell GoC activity modulated during step cycle. Top panels: Black regions show inferred events (size indicates number of events), aligned to each instance of lift or push (different rows). Scaled image in background shows paw height (left panel) or lateral velocity (right, colour scale for relative position shown in panel F). Bottom: Event probability (black) aligned to lift or push, averaged

across all instances ('trials'), and corresponding limb kinematics (blue). Shuffled control (red) was obtained by shuffling events for each 'trial' (within the same 2s block) before averaging.
(D-F) Examples of significantly modulated cells in Crus I from 3 example animals. Total number of significantly modulated cells were n=5/23, n=4/17, n=3/10 in these animals, respectively.

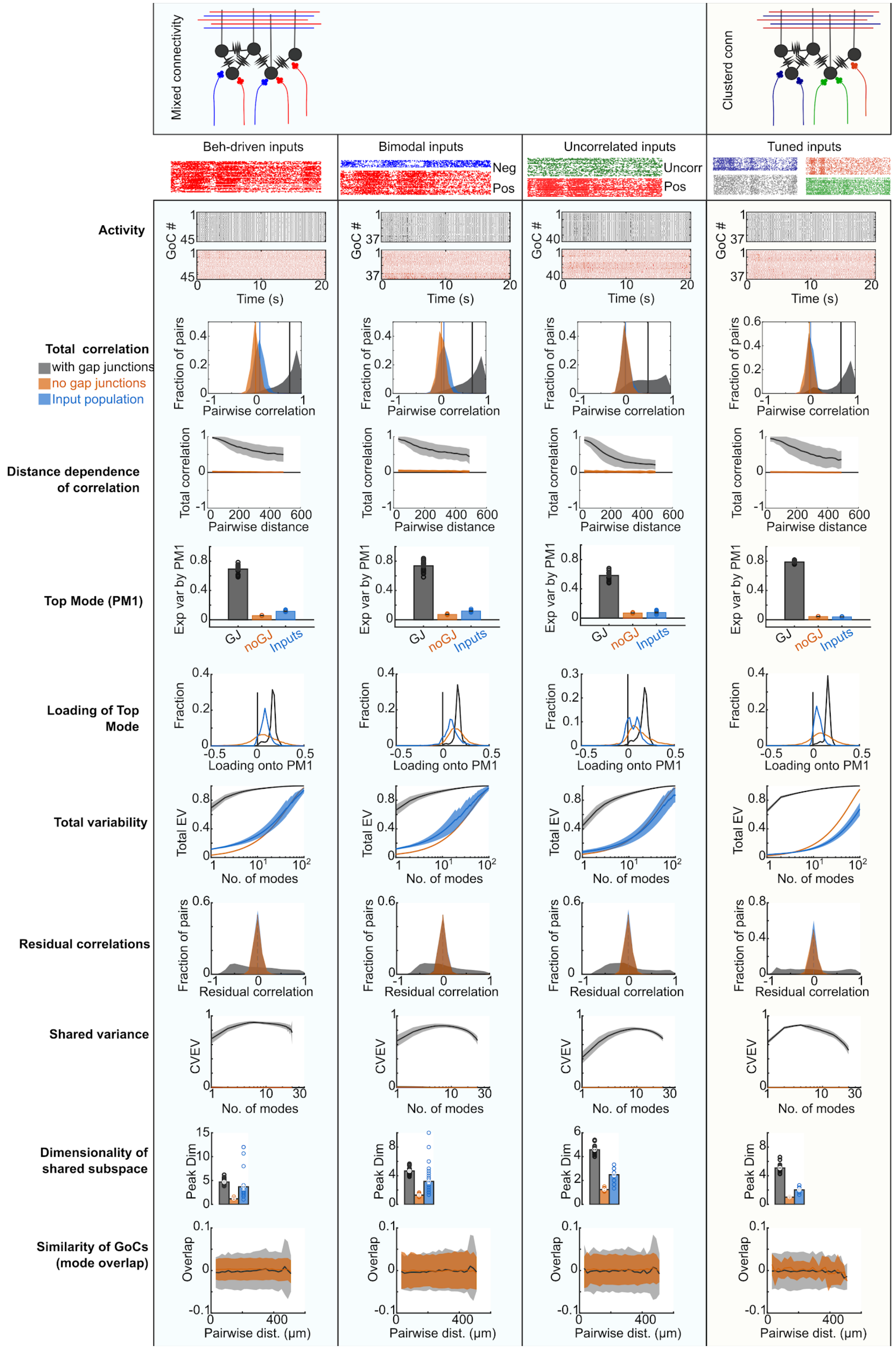

Mixed connectivity
Clusterd conn
Beh-driven inputs
Bimodal inputs
Neg
Pos
Uncorrelated inputs
Uncorr
Pos
Tuned inputs
Activity
GoC #
Time (s)
Total correlation
with gap junctions
no gap junctions
Input population
Fraction of pairs
Pairwise correlation
Distance dependence of correlation
Total correlation
Pairwise distance
Top Mode (PM1)
Exp var by PM1
GJ
noGJ
Inputs
Loading of Top Mode
Fraction
Loading onto PM1
Total variability
Total EV
No. of modes
Residual correlations
Fraction of pairs
Residual correlation
Shared variance
CVEV
No. of modes
Dimensionality of shared subspace
Peak Dim
Similarity of GoCs (mode overlap)
Overlap
Pairwise dist. (µm)

Figure S9: Robustness of population activity in electrically coupled Golgi cell circuit model to spatiotemporal structure of excitatory synaptic input. Related to Figure 8.

Top row: schematic of electrically coupled Golgi cell (GoC) circuit model for spatially mixed (left) and clustered (right) excitatory inputs with example input spike trains below for inputs that are positively (pos, red) and negatively (Neg, blue) modulated by behaviors (Beh). Column 1-3 have random input connectivity, while column 4 has mossy fibre (MF) inputs tuned to specific behaviours restricted to sagittal “zones” , leading to clustered connectivity and higher local input correlations. Example network activity is shown for each of the 4 columns, for GoCs with gap junctions (GJ, black) and without gap junctions (no GJ, orange). Subsequent rows show different measures (pairwise correlations, population modes, dimensionality) of GoC population activity with gap junctions (black) and without gap junctions (orange), as well as of input populations (blue). GoC network activity had a dominant PM1 (top mode) and few differential modes for all the types of inputs tested in electrically coupled circuits. But when GJs were removed the population activity became similar to that of the input. Explained variance (EV), cross validated explained variance (CVEV), dimensionally (Dim), dot product of GoC loadings (Overlap).

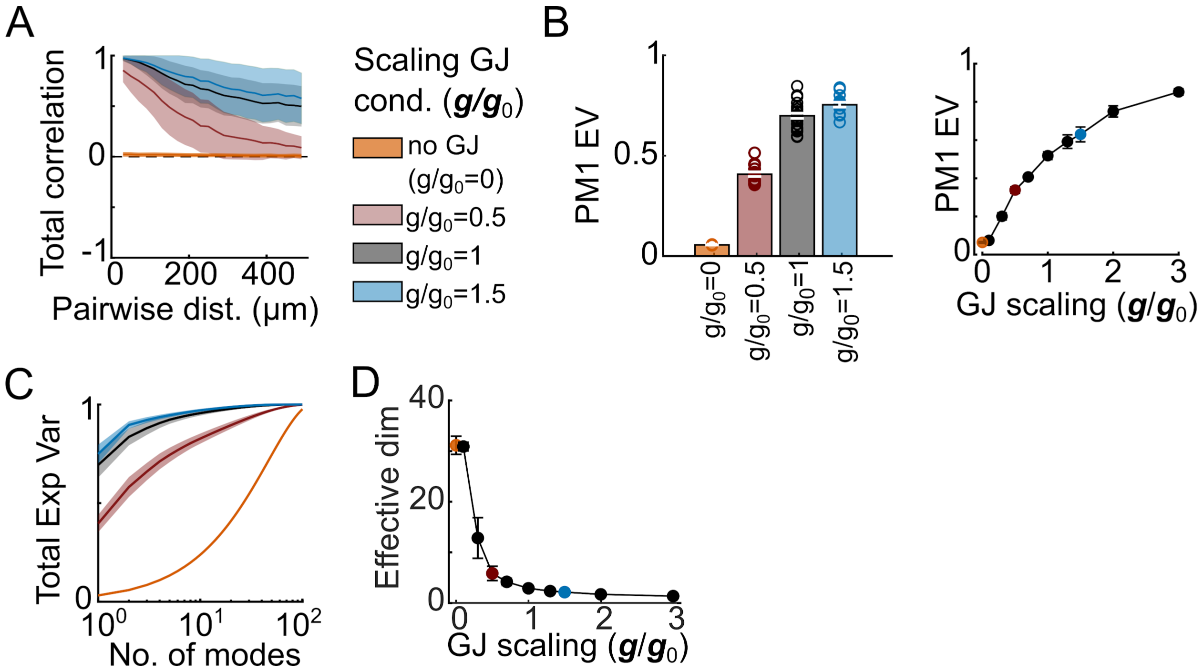

Figure S10: Properties of Golgi cell circuit models with different electrical coupling strengths. Related to Figure 8.

(A) Distance dependence of Golgi cell pairwise correlations for different gap junction (GJ) conductance values (g), expressed in relation to the experimentally measured mean value of 0.9 nS per GJ (g_0): no GJ (orange), decreased by 50% (magenta), no scaling (black), and increased by 50% (blue).

(B) Left: Explained variance (EV) of PM1 (within 'imaging' subvolume) as a function of different GJ conductance strengths. Scatter denotes individual simulations with different input levels, bar and error bar denote mean±SEM. Right: PM1 EV (mean±SEM) for different GJ conductance values (colours of symbols denote values).

(C)Total explained variance as a function of number of population modes, for the four GJ conductance values.

(D) Effective dimensionality (characterising distribution of total variability; mean±SEM) for different GJ conductances.

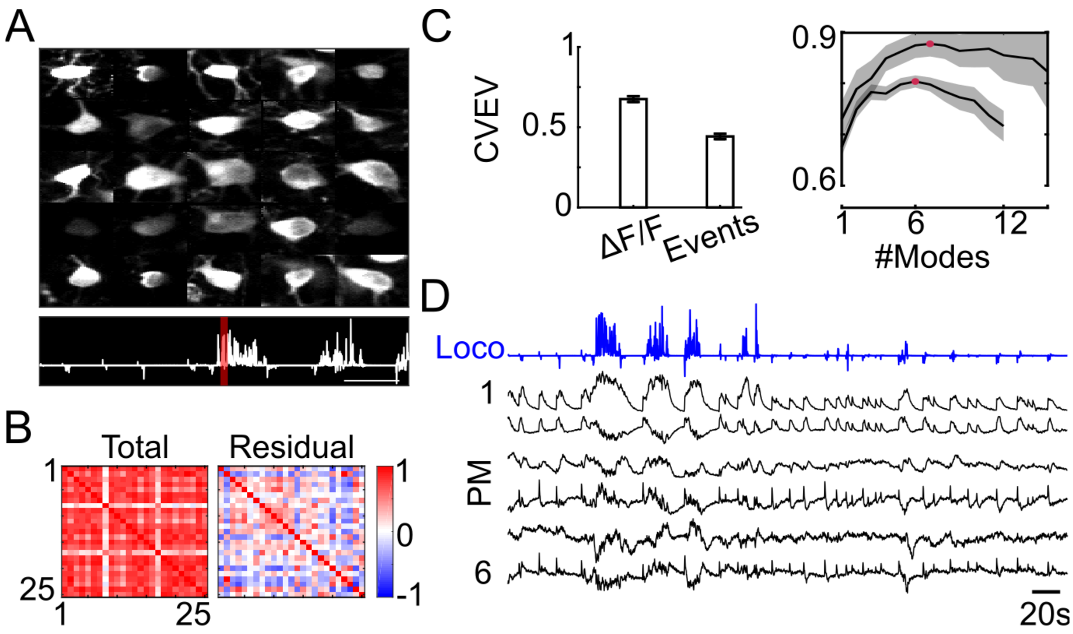

Figure S11: Example sessions with real time 3D movement correction. Related to STAR methods

(A) Mean image (40 frames) of simultaneously recorded Golgi cells (top) with locomotion (bottom) for example session. Scale bar denotes 20 s. Red bar indicates the time period used to create the mean images. This session was recorded with real time 3D motion correction during imaging. This involved tracking a red fluorescent bead in the tissue and correcting the imaging for brain movement. Post-hoc movement correction was applied per imaging patch to correct for the small remaining movements.

(B) Total and residual correlations for the same GoC population as A.

(C) Cross-validated explained variance (CVEV) for (left) first population mode (PM1), and relationship between explained variance and increasing number of modes (right) (n=2 sessions/N=2 animals). Left: Bar and error bar denotes mean ± SEM of explained variance of PM1 for ΔF/F and events. Right: Black line indicates mean, shaded area indicates standard error. Red marker shows the peak of the curve. The estimated shared dimensionality of the two example populations are 6 and 7.

(D) Activity along the top 6 population modes (PM, black), aligned with locomotion (blue).
